# Efficacy and safety analysis of treatment in patients with EGFR-mutated advanced NSCLC who progressed on TKIs: a systematic review and meta-analysis

**DOI:** 10.3389/fimmu.2025.1673115

**Published:** 2025-10-28

**Authors:** Shuang Zhang, Rixin Li, Heran Cui, Hui Li

**Affiliations:** ^1^ Department of Thoracic Oncology, Clinical Research Big Data Center, Jilin Cancer Hospital, Changchun, China; ^2^ Biobank, Jilin Cancer Hospital, Changchun, China

**Keywords:** EGFR-mutated NSCLC, immunotherapy, network meta-analysis, efficacy, safety

## Abstract

**Background:**

The treatment of patients with advanced epidermal growth factor receptor (EGFR)-mutated non-small-cell lung cancer (NSCLC) whose disease progresses after tyrosine-kinase inhibitors (TKIs) treatment has become a research hotspot.

**Objective:**

To identify effective and safe treatment options for patients with EGFR-mutated advanced NSCLC who progressed on TKIs.

**Methods:**

We searched databases including PubMed, Cochrane Library, and major international conference abstracts (2018–2023) to identify phase II/III randomized controlled trials (RCTs) and single-arm studies of EGFR-mutated advanced NSCLC post-TKI progression from April 2018 to June 2024. Outcomes included progression-free survival (PFS), overall survival (OS), objective response rate (ORR), and grade ≥3 adverse events (AEs), treatment-related AEs (TRAEs), and TRAE-related deaths. Bayesian network meta-analysis and individual patient data (IPD) meta-analysis were performed to compare treatment efficacy and safety.

**Results:**

This meta-analysis included randomized controlled trials (RCTs) and 5 single-arm phase 2 trials (3116 patients) evaluating 7 treatment regimens for EGFR-mutated advanced NSCLC post-TKI progression. In the network meta-analysis (NMA), amivantamab plus lazertinib plus chemotherapy (amiva-lazer-chemo) yielded the highest PFS (surface under the cumulative ranking curve [SUCRA]: 0.88; hazard ratio [HR] vs chemotherapy, 0.44; 95% CI, 0.32-0.61), followed by AK112 plus chemotherapy (SUCRA: 0.79; HR, 0.46; 95% CI, 0.32-0.67). All regimens significantly improved PFS compared with chemotherapy alone. Amivantamab plus chemotherapy ranked highest for ORR (SUCRA: 0.82; odds ratios [OR] vs chemotherapy, 3.16; 95% CI, 1.09-9.41). Amiva-lazer-chemo had the highest grade ≥3 AE incidence. IPD analysis confirmed superior PFS for amiva-lazer-chemo (median, 8.45 months; 95% CI, 7.02-9.26; HR vs chemotherapy, 0.47; 95% CI, 0.40-0.55; P <.001). Moderate ORR heterogeneity (I² = 52.2%) and high AE heterogeneity (I² = 79.5%-92.1%) were noted.

**Conclusion:**

In this meta-analysis of patients with TKI-resistant EGFR-mutated advanced NSCLC, the amiva-lazer-chemo regimen was associated with longer PFS at both the study level and individual patient level. Combination therapy with anti-angiogenic agents also represents a viable treatment strategy for this patient population.

**Systematic Review Registration:**

https://www.crd.york.ac.uk/PROSPERO/, identifier CRD42024565403.

## Introduction

The discovery of driver genes and the development of molecularly targeted drugs have revolutionized the treatment of non-small-cell lung cancer (NSCLC), bringing significant survival benefits to patients. Epidermal growth factor receptor (EGFR) mutations are the most common driver gene in patients with NSCLC. Approximately, 15-25% of Caucasian (1, 2) and 40-55% of Asian patients with NSCLC (3, 4) harbor EGFR mutations. Among them, EGFR exon 19 deletion and EGFR L858R point are common and sensitive to EGFR tyrosine-kinase inhibitors (TKIs). EGFR-TKI has become the standard first-line treatment option for EGFR-mutated NSCLC (5). The median progression-free survival (PFS) was 10–14 months with first-generation or second-generation TKI (6–8) and 18.9 months with third-generation EGFR-TKIs for patients with EGFR mutant advanced NSCLC in first-line treatment (9). Although EGFR-TKI can bring benefits to EGFR-mutant NSCLC, drug resistance is inevitable for the vast majority of patients. In order to more effectively manage the treatment of TKI resistance, researchers have also carried out a multi-dimensional exploration.

Platinum-based chemotherapy is the standard treatment option for EGFR mutant advanced patients with NSCLC who do not have a drug therapeutic target after EGFR-TKI resistance (9). However, the efficacy is very limited, with 30% of objective response rate (ORR) and about 5 months of median PFS (10–13). Immunotherapy has become the standard treatment option for EGFR wild-type patients with NSCLC. The efficacy and safety of immune-based protocols in EGFR-TKI resistant patients has also become an important research direction. However, the effect of immune monotherapy on EGFR-TKI resistant patients with NSCLC is very limited, and the results of studies on combination immunotherapy for those patients are also inconsistent. CheckMate 722 (14) and KEYNOTE789 research (15) suggested that neither nivolumab plus chemotherapy nor pembrolizumab plus chemotherapy showed significant improvement in PFS compared to chemotherapy. In ORIENT-31 study, an improved PFS with sintilimab plus chemotherapy vs chemotherapy was apparent specifically (hazard ratios HR, 0.72; 95% CI: 0.55-0.94), but there was no significant improvement in overall survival (OS) (16). Preclinical studies have found that inhibiting VEGF signaling increases T cell infiltration, promotes dendritic cell maturation, attenuating immunosuppressive cell activity, and plays a synergistic role with immunotherapy (17–19). Adding anti-angiogenic drugs to immunotherapy and chemotherapy showed encouraging results. In subgroup analysis of EGFR-sensitive mutations in the IMpower150 study (20), atezolizumab plus bevacizumab plus carboplatin plus paclitaxel (ABCP) regimen significantly improved PFS compared to bevacizumab plus carboplatin plus paclitaxel (BCP) regimen (HR, 0.41; 95% CI: 0.32–0.75). However, the IMpower151 study from China in EGFR/ALK-TKI resistant patients with NSCLC, did not confirm that the ABCP regimen significantly improved PFS compared to BCP (21). The APPLE study from Japan compared the efficacy of atezolizumab plus bevacizumab and chemotherapy with atezolizumab plus chemotherapy. Although this study did not suggest that the atezolizumab, carboplatin plus pemetrexed, and Bevacizumab (APPB) significantly improved PFS in the overall population compared to atezolizumab plus carboplatin with pemetrexed (APP), in the TKI resistant subgroup, APPB achieved a significant benefit in PFS compared with APP (median, 9.7 vs 5.8 months; stratified HR, 0.67; 95% CI, 0.46 to 0.98) (22). It suggested that these studies on the efficacy and safety of immunotherapy-based regimens for EGFR-TKI resistant patients not only have inconsistent conclusions, but also have different control regimens in different studies, which adds to the difficulty of evaluating these regimens. Treatment options after TKI resistance remain challenging. Therefore, it is feasible to evaluate the efficacy and safety of different regimens by conducting meta-analysis that can be indirectly compared, and select the best available treatment regimens for TKI resistant patients.

Recently, the development of bispecific antibodies is also changing the treatment pattern of NSCLC. Ivonescimab (AK112), a bispecific antibody to PD-1 and VEGF, had a significantly favorable safety profile when combined with chemotherapy. In the HARMONi-A study, the median PFS of ivonescimab plus chemotherapy was 7.1 months, which was 4.8 months longer than that of chemotherapy (23). In addition, EGFR-MET dual antibody amivantamab was designed to target MET amplification, a common resistance mechanism of EGFR-TKIs. The phase 3 study (MARIPOSA-2) was also conducted in TKI-resistant NSCLC. This study found that amivantamab-chemotherapy (amiva-chemo) or amivantamab-lazertinib (a third-generation TKI)- chemotherapy (amiva-lazer-chemo) significantly improved PFS in patients with osimertinib-resistant EGFR-mutated NSCLC compared to chemotherapy (24). Whether this dual antibody-based regimen offers an advantage in efficacy and safety over immune-based treatment is lacking in EGFR-TKI resistant patients with NSCLC.

The current treatment options for EGFR-TKI-resistant patients include chemotherapy, anti-vascular drugs plus chemotherapy, immunotherapy-based regimens, dual specificity antibodies-based regimens, etc. However, which regimen is the best choice for EGFR-TKI-treated patients is still uncertain. Although several meta-analyses have evaluated the efficacy and safety of treatment options for TKI-resistant patients with NSCLC, these studies did not include the trails of dual specificity antibodies (25–27). In addition, these meta-analyses were based on the study level, and no individual patient level analysis was performed. Therefore, we included randomized controlled trials (RCTs) and single-arm phase 2 studies, and conducted a meta-analysis to compare the efficacy and safety of these regimens at study level and individual patient level to determine the optimal treatment option for EGFR-TKI-resistant patients with NSCLC.

## Methods

This meta-analysis was performed following Preferred Reporting Items for Systematic Reviews and Meta-Analyses (PRISMA) guidelines, and the protocol was prospectively registered in International Prospective Register of Systematic Reviews (PROSPERO), CRD42024565403.

### Data sources and searches

We searched PubMed and the Cochrane Library for English-language articles published between April 19, 2018, and June 20, 2024, using the combined terms: “NSCLC,” “EGFR,” “resistant,” “immunotherapy,” “chemotherapy,” and “bispecific antibodies” Abstracts from major international conferences—including ASCO, ESMO, WCLC, AACR, and ELCC—were also reviewed (2018–2023). Additionally, reference lists of included studies were manually screened. The full search strategy is provided in **Supplementary Materials** (**Supplementary Table S2**).

### Study selection

**Figure 1:**
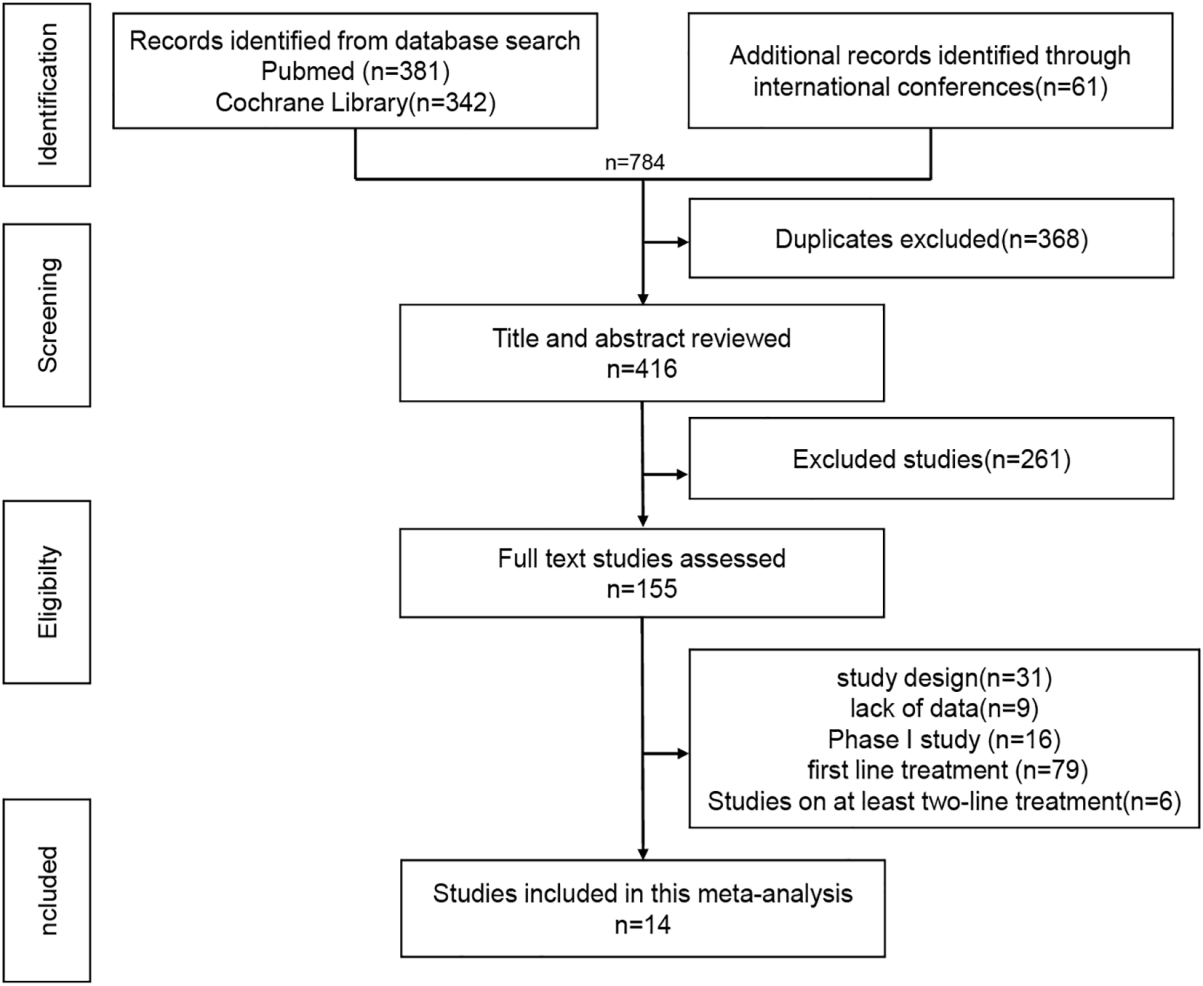
PRISMA flowchart.

The inclusion criteria were as follows: EGFR-mutated advanced NSCLC; patients had progressed after at least one EGFR-TKI treatment; patients with T790M mutation who had received the first- or second-generation EGFR-TKI treatment should receive the third-generation TKI treatment; patients who had not received systemic chemotherapy; Phase III or phase II study; Efficacy and safety related data were available; The exclusion criteria included: retrospective or real-world studies, Phase I studies, sufficient data could not be obtained for meta-analysis. The literature screening process is shown in **Figure 1**.

### Data extraction and quality assessment

Literature screening and data extraction were independently conducted by two authors (SZ and RL) using a standardized form, following Cochrane Collaboration guidelines. Disagreements were resolved through discussion or, if needed, consultation with a third author (HC). Extracted data included study characteristics (publication year, design, phase, sample size, country), patient features (EGFR mutation type [e.g., L858R, exon 19 deletion], T790M status, prior TKI generation, brain metastases), and outcomes (PFS, OS, ORR, HRs with 95% CIs). Safety outcomes included grade ≥3 AEs, TRAEs, grade ≥3 TRAEs, and TRAE-related deaths. When data was incomplete, **Supplementary Materials** were reviewed and study authors contacted. Risk of bias was assessed using the Cochrane Risk of Bias 2 (RoB 2) tool for RCTs and the adapted methodological index for non-randomized studies (MINORS) criteria for single-arm phase II trials. Two reviewers independently performed the assessment, with discrepancies resolved by a third reviewer.

### Statistical analysis

A Bayesian network meta-analysis (NMA) was performed using R (v4.3.1) with the *multinma* and *gemtc* packages. The primary outcome was PFS; secondary outcomes included OS, ORR, and safety outcomes (grade ≥3 AE, TRAEs, grade ≥3 TRAEs, and TRAE-related deaths). Safety analyses were performed to evaluate the incidence of adverse events (AEs) across treatment arms. For zero events in safety analyses, we applied a continuity correction of 0.5, adding 0.5 to cells with zero events to avoid computational instability and ensure stable estimates. NMA was conducted within a Bayesian framework via Markov chain Monte Carlo (MCMC) methods. Non-informative priors were applied; heterogeneity was modeled using a uniform (0–5) prior, the uniform (0-5) prior was selected based on common practice in Bayesian network meta-analyses, as this range reasonably encompasses typical values of the standard deviation of heterogeneity (τ), typically between 0 and 5, reflecting the potential magnitude of between-trial variability. Three MCMC chains were run for 10,000 iterations each, including 2,000 burn-in iterations. To assess model fit and consistency, we compared the goodness of fit between consistency and inconsistency models using DIC values. Convergence was evaluated using Gelman-Rubin diagnostic plots, and trace and posterior density plots for treatment effect parameters were generated to further validate model stability. Node-splitting tests were performed to comprehensively evaluate network consistency. Treatment effects were expressed as hazard ratios (HRs) or odds ratios (ORs) with 95% credible intervals (CrI). Surface under the cumulative ranking curve (SUCRA) values were used for treatment ranking, with higher SUCRA indicating better performance. Ranking probabilities assessed the likelihood of each treatment being among the best.

For individual patient data (IPD) meta-analysis, IPD were reconstructed from Kaplan-Meier curves using the *IPDfromKM* (28) package in R. Median PFS was estimated and pooled Kaplan-Meier curves were generated for each regimen. Group differences were compared using log-rank tests (P < 0.05 for significance). A one-stage approach was applied to the reconstructed IPD using the *frailtypack* package to build a random-effects shared frailty Cox model. Non-parametric penalized likelihood estimation and spline-smoothed baseline hazards were used to account for within- and between-study heterogeneity.

Heterogeneity in pairwise meta-analyses was assessed using the I² statistic and Cochran’s Q test, with I² > 50% or P < 0.1 indicating significant heterogeneity. Subgroup analyses were conducted to explore potential sources of heterogeneity, including brain metastasis status, EGFR mutation subtype, and prior TKI regimen.

## Results

### Systematic review and characteristics of all trials

A total of 14 studies were included, comprising 5 single-arm trials (21, 29–32) and 9 RCTs (14–16, 21–24, 33, 34), enrolling 3,116 patients across 7 treatment regimens: chemotherapy (chemo), ICI - chemotherapy (ICI-chemo), ICI-chemo - anti-angiogenic therapy (ICI-chemo-antiangio), chemotherapy - anti-angiogenesis (chemo-antiangio), AK112 - chemo (AK112-chemo), amivantamab - chemo (amiva-chemo), and amivantamab - lazertinib - chemo (amiva-lazer-chemo) (**Supplementary Table S1**).

### Network meta-analyses for outcomes

**Figure 2:**
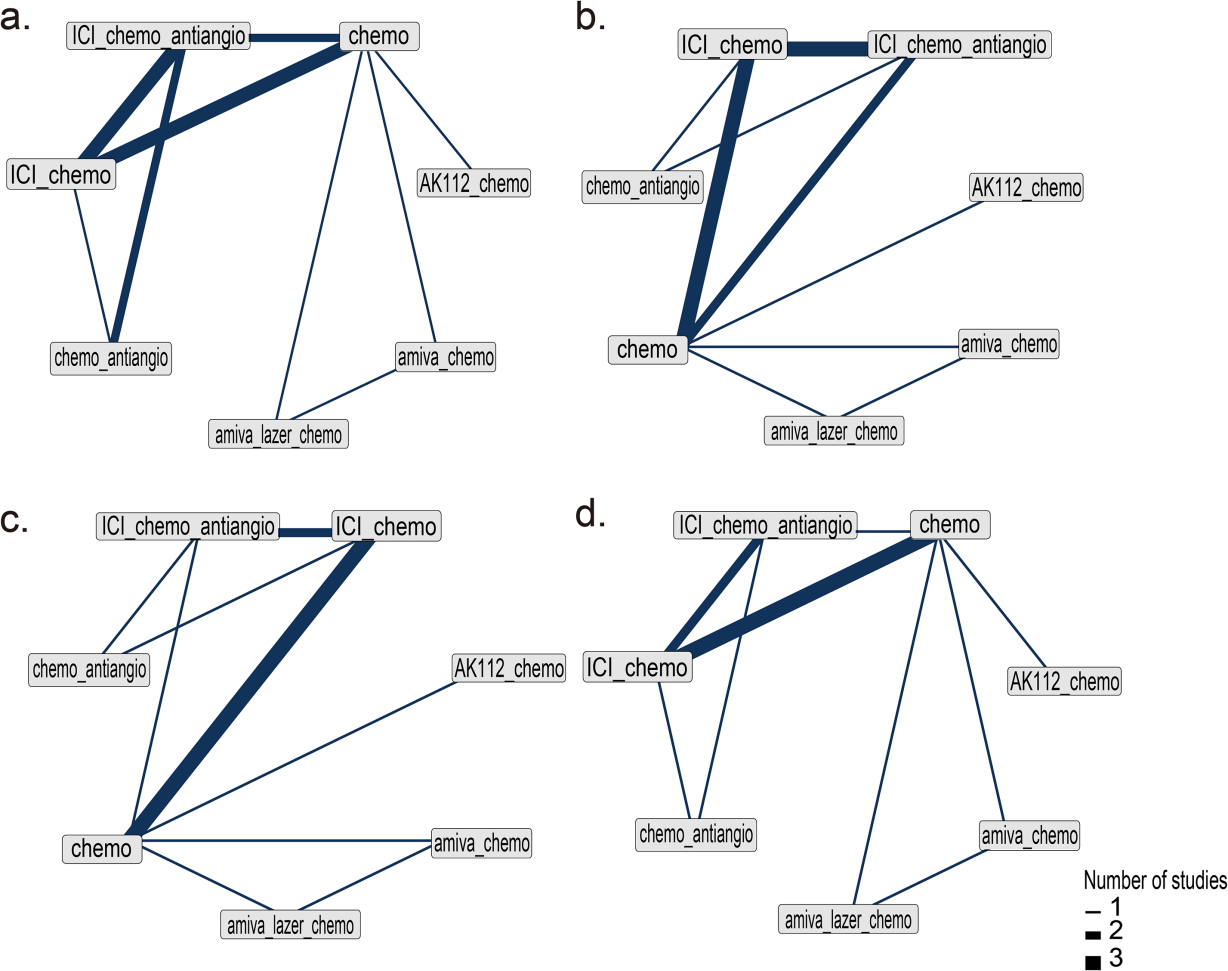
Network diagram of network meta-analysis. **(a)** Progression-free survival (PFS), **(b)** Overall survival (OS), **(c)** Objective response rate (ORR), **(d)** Grade 3 or higher adverse events (AEs). The analysis is based solely on randomized controlled trials (RCTs).

**Figure 3:**
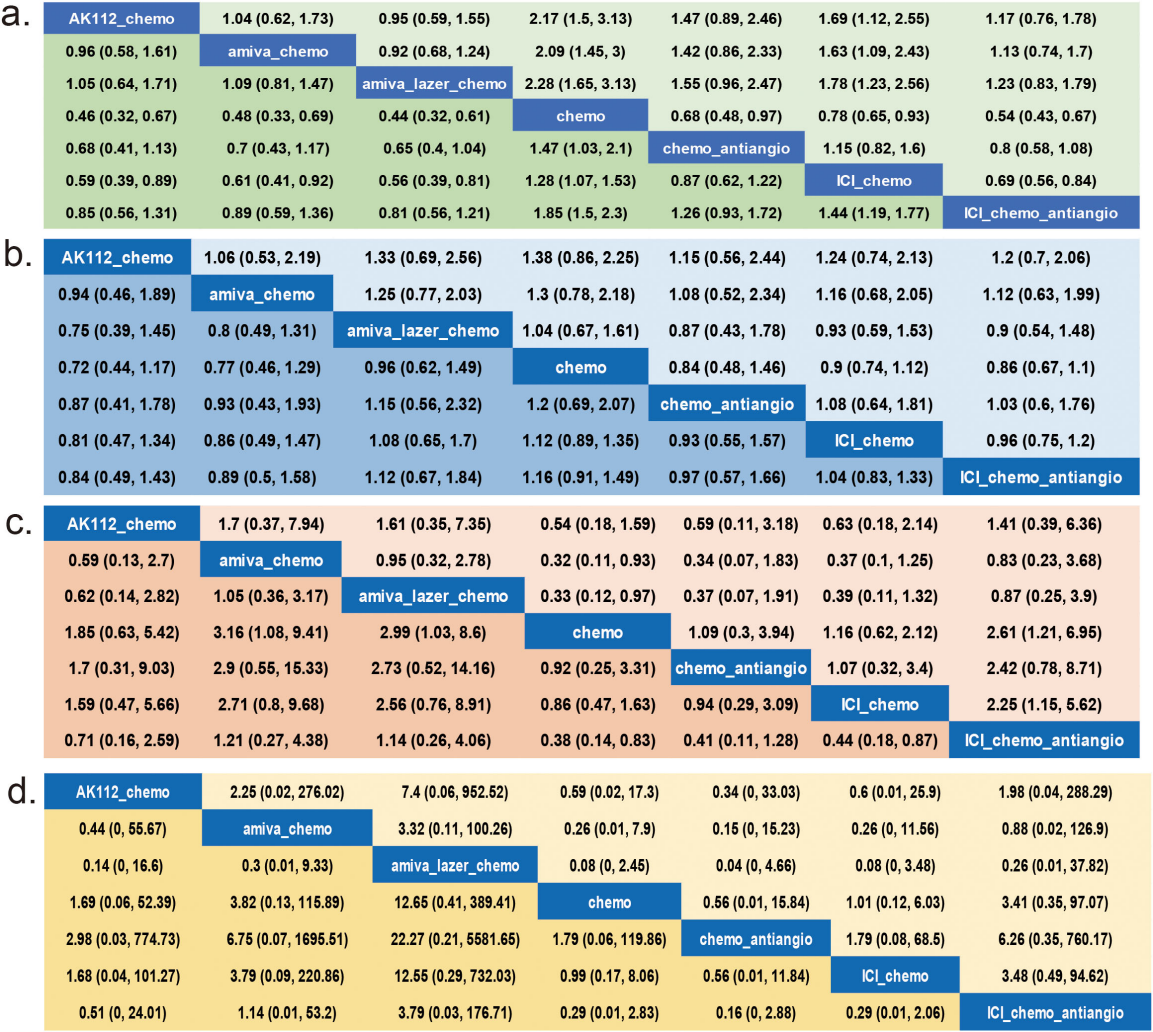
League Table for the network meta-analysis. **(a)** Progression-free survival (PFS), **(b)** Overall survival (OS), **(c)** Objective response rate (ORR), **(d)** Grade 3 or higher adverse events (AEs). The analysis is based solely on randomized controlled trials (RCTs).

Nine RCTs (14–16, 21–24, 33, 34) (n = 2,850) were included in the network meta-analysis. All trials reported PFS; 7 reported OS (14–16, 22–24, 33, 34), 6 reported ORR (14–16, 22–24, 33, 34), and 7 reported AEs (14–16, 22–24, 33, 34) (**Figures 2a–d**).All regimens showed significantly improved PFS vs. chemo. The best-performing were amiva-lazer-chemo (HR, 0.44; 95% CrI, 0.32–0.61), AK112-chemo (HR, 0.46; 95% CrI, 0.32–0.67), and amiva-chemo (HR, 0.48; 95% CrI, 0.33–0.69) (**Figure 3a**). Compared with ICI-chemo, these regimens also showed superior PFS (HR range, 0.56–0.69). No significant differences were observed among these top regimens or between ICI-chemo and chemo-antiangio (HR, 0.80; 95% CrI, 0.58–1.08)(**Figure 3a**). No significant differences were found in OS across treatment arms (**Figure 3b**). Amiva-chemo (OR, 3.16; 95% CrI, 1.09–9.41), amiva-lazer-chemo (OR, 2.99; 95% CrI, 1.03–8.60), and ICI-chemo-antiangio (OR, 2.61; 95% CrI, 1.21–6.95) had significantly ORR than chemotherapy. ICI-chemo-antiangio also showed higher ORR than ICI-chemo (OR, 2.25; 95% CrI, 1.15–5.62) (**Figure 3c**). Grade ≥3 AEs were significantly higher with amiva-lazer-chemo vs. chemo, chemo-antiangio, ICI-chemo, and ICI-chemo-antiangio. No significant differences in grade ≥3 TRAEs were observed across regimens (**Figure 3d**).

### Rank probability and inconsistency assessment

**Figure 4:**
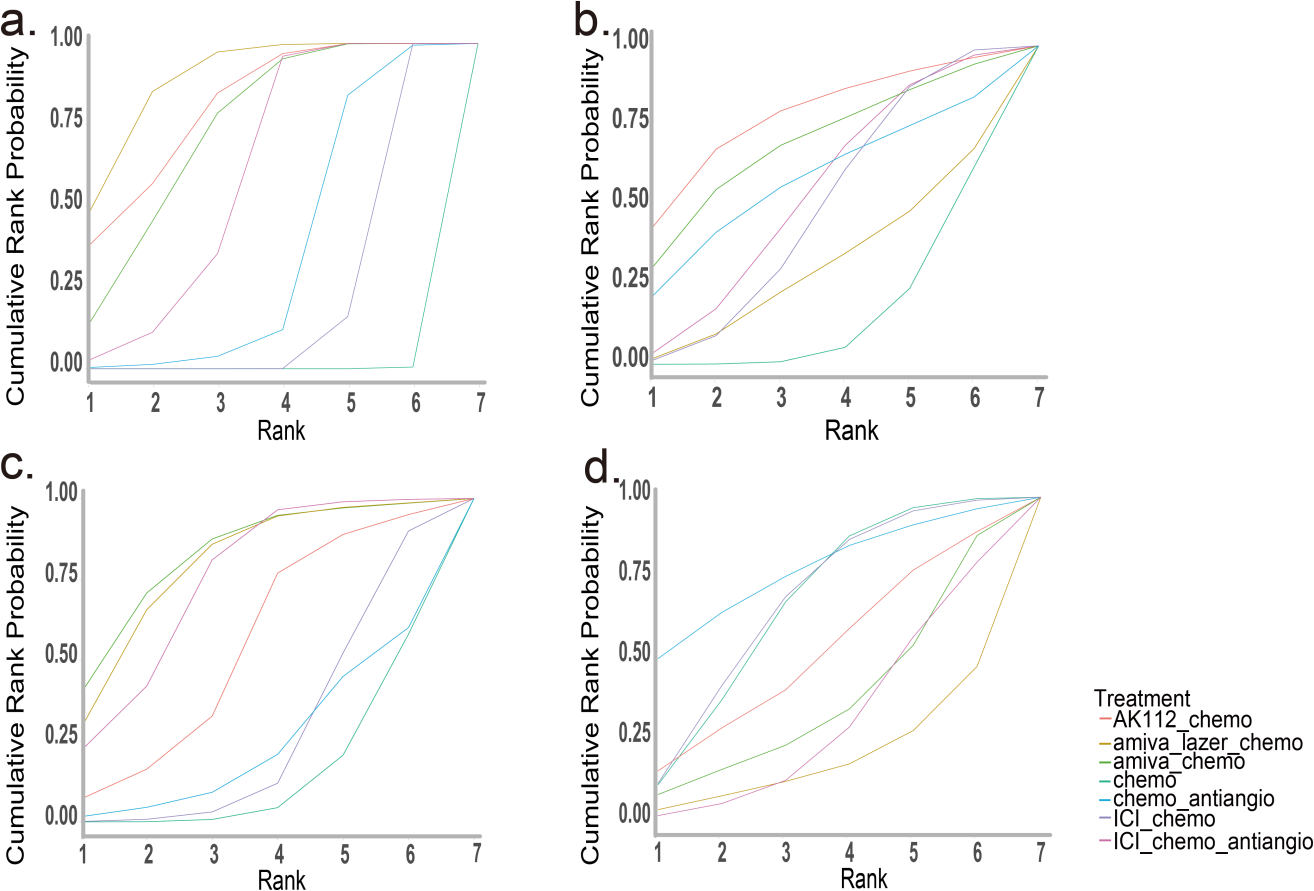
SUCRA for the network meta-analysis. **(a)** Progression-free survival (PFS), **(b)** Overall survival (OS), **(c)** Objective response rate (ORR), **(d)** Grade 3 or higher adverse events (AEs). The analysis is based solely on randomized controlled trials (RCTs).

Bayesian SUCRA ranking (**Figure 4**) showed amiva-lazer-chemo had the highest probability for PFS (0.88), followed by AK112-chemo (0.79) and amiva-chemo (0.72). For OS, AK112-chemo ranked first (0.77). Amiva-chemo ranked highest for ORR (0.82), while amiva-lazer-chemo had the highest risk of grade ≥3 AEs.

### Individual patient data meta-analysis for PFS

A one-stage IPD meta-analysis was performed using a shared frailty Cox model to assess PFS, incorporating study type (single-arm or RCT) as a clustering factor. The baseline hazard was modeled via cubic spline interpolation with 10 nodes and a smoothing parameter of 10,000.

**Figure 5:**
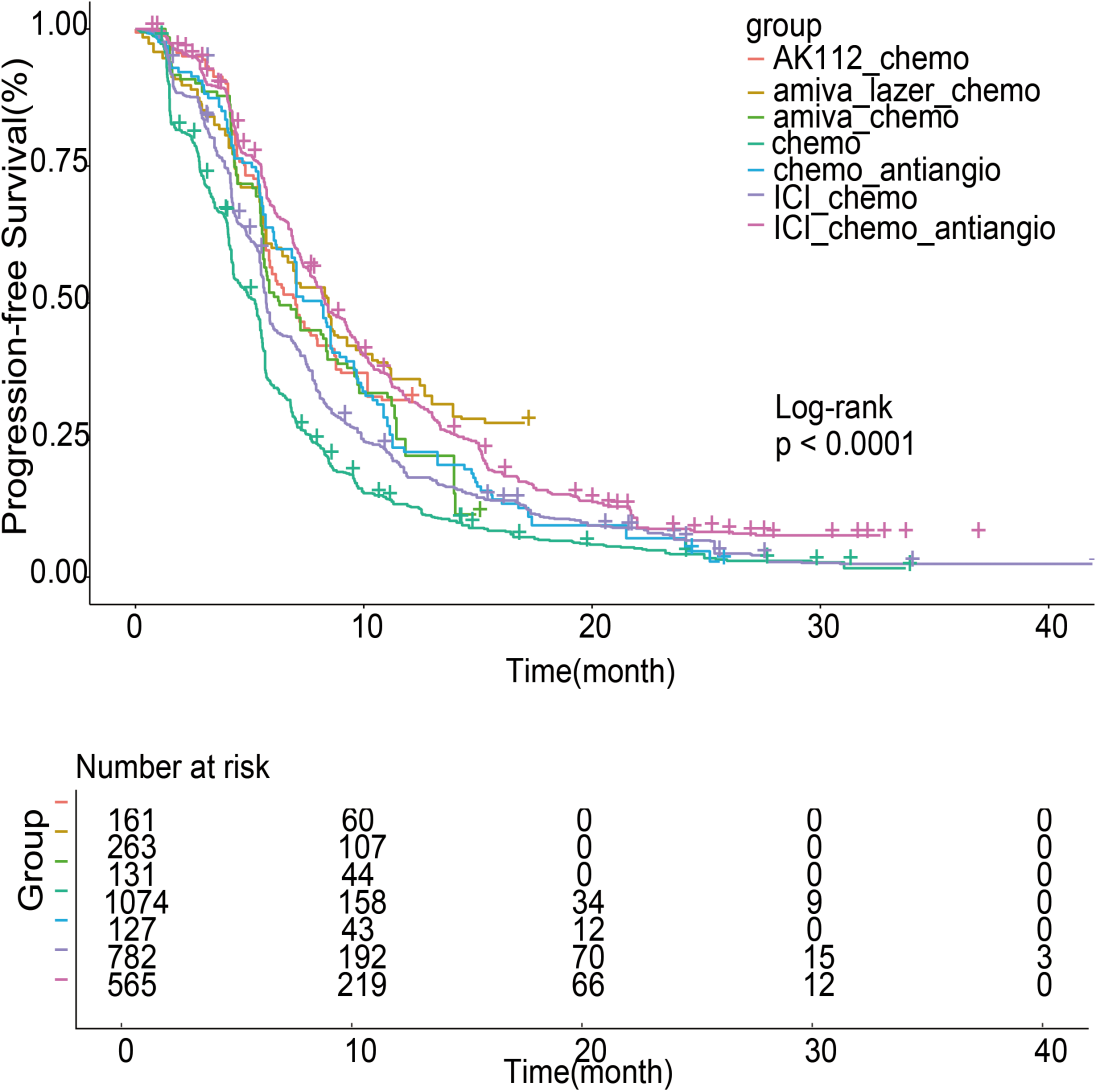
Survival curves of progression-free survival (PFS) for patients after individual patient data (IPD) reconstruction. The analysis used randomized controlled trials (RCTs) and single-arm studies.

Pooled Kaplan-Meier curves (**Figure 5**) showed significant PFS differences across seven treatment groups (log-rank test, P < 0.001). Median PFS was longest for amiva-lazer-chemo (8.45 months; 95% CI, 7.02–9.26), followed by ICI-chemo-antiangio (8.30 months; 95% CI, 7.88-9.27), chemo-antiangio (8.15; 95% CI, 6.98-8.95), AK112-chemo (7.01 months; 95% CI, 5.89-8.59), amiva-chemo (6.31 months; 95% CI, 5.65-8.41), ICI-chemo (5.73 months; 95% CI, 5.61-5.90), and chemotherapy alone (5.24 months; 95% CI, 4.67-5.46).

Using chemo-antiangio as the reference, amiva-lazer-chemo significantly improved PFS (HR, 0.73; 95% CI, 0.58–0.91; P < 0.001). ICI-chemo-antiangio (HR, 0.82; 95% CI, 0.68–1.00; P = 0.055) and AK112-chemo (HR, 0.79; 95% CI, 0.61–1.01; P = 0.064) showed non-significant trends toward benefit. Amiva-chemo (HR, 1.05; 95% CI, 0.81-1.35; *P* = 0.72) and ICI-chemo (HR, 1.17; 95% CI, 0.96-1.41; *P* = 0.12) were not significantly different. Compared with chemotherapy alone, all combination regimens significantly improved PFS: amiva-lazer-chemo (HR, 0.47; 95% CI, 0.40-0.55; *P* < 0.001), ICI-chemo-antiangio (HR, 0.53; 95% CI, 0.48-0.59; *P* < 0.001), AK112-chemo (HR, 0.49; 95% CI, 0.41-0.59; *P* < 0.001), amiva-chemo (HR, 0.67; 95% CI, 0.55-0.82; *P* < 0.001), ICI-chemo (HR, 0.75; 95% CI, 0.68-0.82; *P* < 0.001), and chemo-antiangio (HR, 0.64; 95% CI, 0.53-0.78; *P* < 0.001), all with P < 0.001.

### Risk of bias and data quality

No significant heterogeneity was found in PFS and OS outcomes (**Supplementary Figures S1**, **S2**). Moderate heterogeneity was noted for ORR between ICI-chemo-antiangio and ICI-chemo (I² = 52.2%; **Supplementary Figure S3**). In contrast, high heterogeneity was observed in AEs comparisons: ICI-chemo-antiangio vs chemotherapy (I² = 92.1%), ICI-chemo-antiangio vs ICI-chemo (I² = 83.5%), and ICI-chemo vs chemotherapy (I² = 79.5%) (**Supplementary Figure S4**). Most RCTs had low to moderate risk of bias per Cochrane RoB 2 (**Supplementary Figures S5**, **S6**), while single-arm studies were rated low risk using MINORS (**Supplementary Table S3**). The network meta-analysis demonstrated robust convergence, with Gelman-Rubin diagnostic plots indicating shrink factors approaching 1 (**Supplementary Figure S12**). Trace and posterior density plots for treatment effect parameters further confirmed model stability (**Supplementary Figure S11**). Consistency assessment revealed no significant inconsistency, as supported by node-splitting tests and the comparison of DIC values between consistency and inconsistency models (**Supplementary Table S5**). These findings underpin the reliability of the indirect comparisons reported.

### Subgroup Analysis

We conducted subgroup analyses based on EGFR mutation subtype, brain metastasis status, and prior TKI regimen. Compared to chemo, we observed PFS benefits in most subgroups receiving ICI-chemo (**Supplementary Figures S8a**, **S9a**), with the exception of the subgroups with prior 1st/2nd generation TKI and 3rd generation TKI, where the results were reversed (**Supplementary Figure S7**). In the L858R and 19DEL mutation subgroups, both ICI-chemo and ICI-chemo-antiangio showed PFS benefits compared to chemo, with L858R mutation patients exhibiting better PFS than 19DEL patients (**Supplementary Figure S8**). In the T790M+ and T790M- subgroups, both ICI-chemo and ICI-chemo-antiangio demonstrated PFS benefits compared to chemo, with T790M- patients showing better PFS than T790M+ patients (**Supplementary Figure S9**). In the brain metastasis subgroups, ICI-chemo-antiangio provided PFS benefits compared to chemo, with patients without brain metastasis exhibiting better PFS than those with brain metastasis (**Supplementary Figure S10**).

## Discussion

This meta-analysis is the first to evaluate the efficacy and safety of treatment strategies for patients with advanced EGFR-mutated NSCLC after EGFR-TKI progression at both the study level and the patient level. In addition, our study included 7 different therapeutic strategies, including immune-based regimen, double-antibody based regimen, chemo, and chemo-antiangio and so on, which covered the most comprehensive therapeutic strategies for this population at present. Our study indicates that bispecific antibody-based regimens demonstrate superior PFS and ORR compared with chemo or ICI-chemo, both at the study level and the patient level, in patients with EGFR-mutated advanced NSCLC who have progressed after TKI treatment. We also emphasize that these results be clearly labeled as preliminary, with stronger caveats that OS outcomes are unresolved and require further follow-up. The present conclusions are primarily supported by PFS and ORR, and that the long-term survival impact of these regimens, particularly in the context of added toxicity remains to be determined.

The development of bispecific antibodies has emerged as a critical strategy to overcome resistance to EGFR TKIs. Our study represents the first meta-analysis to evaluate the efficacy and safety of treatment regimens incorporating bispecific antibodies in patients with EGFR-mutated advanced NSCLC following TKI resistance. In the network meta-analysis, which included randomized controlled trials, regimens incorporating bispecific antibodies demonstrated significantly longer PFS and higher ORR compared with chemo or ICI-chemo. SUCRA rankings further confirmed the superiority of bispecific antibody-based regimens in PFS and ORR.These findings highlight the clear efficacy advantage of bispecific antibody-based regimens. Previous meta-analyses suggested that ICI-chemo-antiangio was the optimal treatment for TKI-resistant patients (26, 35). Notably, both the AK112-chemo and ICI-chemo-antiangio increased VEGF levels following TKI resistance. However, the bispecific antibody-based regimen targeting PD-1 and VEGF-A (AK112-chemo) demonstrated superior long-term efficacy, while ICI-chemo-antiangio showed better short-term efficacy (higher ORR) but higher toxicity. Our meta-analysis indicates that bispecific antibody-based regimens represent a highly promising research direction for TKI-resistant patients. Given that bispecific antibodies target distinct TKI resistance mechanisms, we did not pool these regimens in the meta-analysis. Currently, most randomized controlled trials in patients with resistance to first- or second-generation TKIs without T790M mutations or to third-generation TKIs have not stratified participants based on resistance mechanisms. Developing bispecific antibodies tailored to specific resistance mechanisms and selecting bispecific antibody-based treatment regimens will be critical to meeting the demands of precision medicine in the post-TKI resistance setting.

Our meta-analysis differs from prior studies by conducting both study-level and IPD meta-analyses. In the context of large-scale randomized controlled trials, IPD-based meta-analyses provide more reliable evidence-based comparisons of treatment efficacy (36, 37). With the advent of IPD reconstruction from survival curves, overcoming data acquisition challenges, patient-level analyses have gained increasing attention. Our IPD analysis incorporated both randomized controlled trials and single-arm studies, pooling data for identical treatment strategies. In the IPD-based analysis, all alternative treatment strategies demonstrated superior PFS compared with standard chemotherapy. Notably, four treatment regimens—ICI-chemo-antiangio, amiva-chemo, ICI-chemo, AK112-chemo—exhibited median PFS values comparable to chemo-antiangio, consistent with the findings of our network meta-analysis.

Compared with chemo-antiangio, amiva-lazer-chemo significantly improved PFS in patients with EGFR-mutated NSCLC following TKI resistance in the IPD analysis. In contrast, the network meta-analysis showed a trend toward PFS benefit for amiva-lazer-chemo but did not reach statistical significance, potentially due to limited sample sizes in both analyses. This discrepancy suggests that chemo-antiangio may be a more appropriate control arm for future studies investigating optimal treatment strategies for EGFR-mutated, TKI-resistant NSCLC.

There are inherent limitations to our studies. First of all, the evaluation of OS in this meta-analysis was inadequate In the ATTLAS study, the APPLE Study, and the ORIENT-31 study, the OS maturity was less than 60% (16, 22, 33). The number of OS events in the MARIPOSA-2 study was less than 30% (24). In the HARMONi-a study, OS data is still not reported (24). As the OS of these studies gradually matures, the results of OS analysis in the meta-analysis may be different. Secondly, compared with other meta-analyses, our study included more treatments, but the published results of some regiments, for example bispecific antibody-based regiments, are limited. It is ongoing that a phase II/III study of PM8002(a bispecific antibody targeting PD-L1 and VEGF) in combination with chemotherapy in patients with EGFR-mutant advanced NSCLC who have failed to EGFR-TKI treatment (NCT05756972). Thirdly, Antibody-drug conjugates (ADCs) is also an important research direction for treatment selection after TKI resistance. However, most of the published studies focused on TKI-resistant patients who received platinum-based chemotherapy. HER3-targeting ADCs and TROP2-targeting ADCs have shown promising efficacy in phase 1 and 2 studies of patients with EGFR-mutated NSCLC who progressed after EGFR -TKI and platinum-based chemotherapy (38–40). Studies of these agents in EGFR-mutated patients with NSCLC who treated only with TKIs are ongoing (NCT06382116; NCT06417814; NCT05338970). Whether ADC can outperform existing treatment options needs to await the results of these studies and a new meta-analysis will be performed to clarify.

## Conclusion

Our meta-analysis suggests that, for patients with EGFR-mutated NSCLC who have progressed on TKI therapy, the amiva-lazer-chemo regimen is the preferred option for delaying disease progression at both the study and individual patient levels. However, the toxicity of this regimen warrants careful consideration. Combination regimens incorporating antiangiogenic therapy, including ICI-chemo-antiangio, chemo-antiangio, and AK112-chemo, also demonstrated superior efficacy compared with standard chemotherapy. Our findings provide critical insights to guide subsequent treatment decisions for EGFR-mutated NSCLC patients with TKI relapse.

## Data Availability

The original contributions presented in the study are included in the article/**Supplementary Material**. Further inquiries can be directed to the corresponding author.

## References

[1] Rosell R Moran T Queralt C Porta R Cardenal F Camps C . Screening for epidermal growth factor receptor mutations in lung cancer. N Engl J Med. (2009) 361:958–67. doi: 10.1056/NEJMoa0904554, PMID: 19692684

[2] Jordan EJ Kim HR Arcila ME Barron D Chakravarty D Gao J . Prospective comprehensive molecular characterization of lung adenocarcinomas for efficient patient matching to approved and emerging therapies. Cancer Discov. (2017) 7:596–609. doi: 10.1158/2159-8290.CD-16-1337, PMID: 28336552 PMC5482929

[3] Liu L Liu J Shao D Deng Q Tang H Liu Z . Comprehensive genomic profiling of lung cancer using a validated panel to explore therapeutic targets in East Asian patients. Cancer Sci. (2017) 108:2487–94. doi: 10.1111/cas.13410, PMID: 28949084 PMC5715245

[4] Wen S Dai L Wang L Wang W Wu D Wang K . Genomic signature of driver genes identified by target next-generation sequencing in chinese non-small cell lung cancer. Oncologist. (2019) 24:e1070–e81. doi: 10.1634/theoncologist.2018-0572, PMID: 30902917 PMC6853120

[5] Riely GJ Wood DE Ettinger DS Aisner DL Akerley W Bauman JR . Non-small cell lung cancer, version 4.2024, NCCN clinical practice guidelines in oncology. J Natl Compr Canc Netw. (2024) 22:249–74. doi: 10.6004/jnccn.2204.0023, PMID: 38754467

[6] Mok TS Wu YL Thongprasert S Yang CH Chu DT Saijo N . Gefitinib or carboplatin-paclitaxel in pulmonary adenocarcinoma. N Engl J Med. (2009) 361:947–57. doi: 10.1056/NEJMoa0810699, PMID: 19692680

[7] Zhou C Wu YL Chen G Feng J Liu XQ Wang C . Erlotinib versus chemotherapy as first-line treatment for patients with advanced EGFR mutation-positive non-small-cell lung cancer (OPTIMAL, CTONG-0802): a multicentre, open-label, randomised, phase 3 study. Lancet Oncol. (2011) 12:735–42. doi: 10.1016/S1470-2045(11)70184-X, PMID: 21783417

[8] Wu YL Cheng Y Zhou X Lee KH Nakagawa K Niho S . Dacomitinib versus gefitinib as first-line treatment for patients with EGFR-mutation-positive non-small-cell lung cancer (ARCHER 1050): a randomised, open-label, phase 3 trial. Lancet Oncol. (2017) 18:1454–66. doi: 10.1016/S1470-2045(17)30608-3, PMID: 28958502

[9] Soria JC Ohe Y Vansteenkiste J Reungwetwattana T Chewaskulyong B Lee KH . Osimertinib in untreated EGFR-mutated advanced non-small-cell lung cancer. N Engl J Med. (2018) 378:113–25. doi: 10.1056/NEJMoa1713137, PMID: 29151359

[10] Wu YL Planchard D Lu S Sun H Yamamoto N Kim DW . Pan-Asian adapted Clinical Practice Guidelines for the management of patients with metastatic non-small-cell lung cancer: a CSCO-ESMO initiative endorsed by JSMO, KSMO, MOS, SSO and TOS. Ann Oncol. (2019) 30:171–210. doi: 10.1093/annonc/mdy554, PMID: 30596843

[11] Soria JC Wu YL Nakagawa K Kim SW Yang JJ Ahn MJ . Gefitinib plus chemotherapy versus placebo plus chemotherapy in EGFR-mutation-positive non-small-cell lung cancer after progression on first-line gefitinib (IMPRESS): a phase 3 randomised trial. Lancet Oncol. (2015) 16:990–8. doi: 10.1016/S1470-2045(15)00121-7, PMID: 26159065

[12] Hendriks LE Kerr KM Menis J Mok TS Nestle U Passaro A . Oncogene-addicted metastatic non-small-cell lung cancer: ESMO Clinical Practice Guideline for diagnosis, treatment and follow-up. Ann Oncol. (2023) 34:339–57. doi: 10.1016/j.annonc.2022.12.009, PMID: 36872130

[13] Ettinger DS Wood DE Aisner DL Akerley W Bauman JR Bharat A . Non-small cell lung cancer, version 3.2022, NCCN clinical practice guidelines in oncology. J Natl Compr Canc Netw. (2022) 20:497–530. doi: 10.6004/jnccn.2022.0025, PMID: 35545176

[14] Mok T Nakagawa K Park K Ohe Y Girard N Kim HR . Nivolumab plus chemotherapy in epidermal growth factor receptor-mutated metastatic non-small-cell lung cancer after disease progression on epidermal growth factor receptor tyrosine kinase inhibitors: final results of checkMate 722. J Clin Oncol. (2024) 42:1252–64. doi: 10.1200/JCO.23.01017, PMID: 38252907 PMC11095864

[15] Yang JC Lee DH Lee JS Fan Y de Marinis F Iwama E . Phase III KEYNOTE-789 study of pemetrexed and platinum with or without pembrolizumab for tyrosine kinase inhibitor–Resistant, EGFR-mutant, metastatic nonsquamous non-small cell lung cancer. J Clin Oncol. (2024) 42:4029–39. doi: 10.1200/JCO.23.02747, PMID: 39173098 PMC11608596

[16] Lu S Wu L Jian H Cheng Y Wang Q Fang J . Sintilimab plus chemotherapy for patients with EGFR-mutated non-squamous non-small-cell lung cancer with disease progression after EGFR tyrosine-kinase inhibitor therapy (ORIENT-31): second interim analysis from a double-blind, randomised, placebo-controlled, phase 3 trial. Lancet Respir Med. (2023) 11:624–36. doi: 10.1016/S2213-2600(23)00135-2, PMID: 37156249

[17] Motz GT Santoro SP Wang LP Garrabrant T Lastra RR Hagemann IS . Tumor endothelium FasL establishes a selective immune barrier promoting tolerance in tumors. Nat Med. (2014) 20:607–15. doi: 10.1038/nm.3541, PMID: 24793239 PMC4060245

[18] Huang Y Goel S Duda DG Fukumura D Jain RK . Vascular normalization as an emerging strategy to enhance cancer immunotherapy. Cancer Res. (2013) 73:2943–8. doi: 10.1158/0008-5472.CAN-12-4354, PMID: 23440426 PMC3655127

[19] Buckanovich RJ Facciabene A Kim S Benencia F Sasaroli D Balint K . Endothelin B receptor mediates the endothelial barrier to T cell homing to tumors and disables immune therapy. Nat Med. (2008) 14:28–36. doi: 10.1038/nm1699, PMID: 18157142

[20] Nogami N Barlesi F Socinski MA Reck M Thomas CA Cappuzzo F . IMpower150 final exploratory analyses for atezolizumab plus bevacizumab and chemotherapy in key NSCLC patient subgroups with EGFR mutations or metastases in the liver or brain. J Thorac Oncol. (2022) 17:309–23. doi: 10.1016/j.jtho.2021.09.014, PMID: 34626838

[21] Zhou C Dong X Chen G Wang Z Wu X Yao Y . Atezolizumab plus bevacizumab and chemotherapy in metastatic nonsquamous NSCLC: the randomized double-blind phase 3 IMpower151 trial. Nat Med. (2025) 31:2375–84. doi: 10.1038/s41591-025-03658-y, PMID: 40379995 PMC12283377

[22] Shiraishi Y Kishimoto J Sugawara S Mizutani H Daga H Azuma K . Atezolizumab and platinum plus pemetrexed with or without bevacizumab for metastatic nonsquamous non-small cell lung cancer: A phase 3 randomized clinical trial. JAMA Oncol. (2024) 10:315–24. doi: 10.1001/jamaoncol.2023.5258, PMID: 38127362 PMC10739077

[23] Fang W Zhao Y Luo Y Yang R Huang Y He Z . Ivonescimab plus chemotherapy in non-small cell lung cancer with EGFR variant: A randomized clinical trial. Jama. (2024) 332:561–70. doi: 10.1001/jama.2024.10613, PMID: 38820549 PMC11337070

[24] Passaro A Wang J Wang Y Lee SH Melosky B Shih JY . Amivantamab plus chemotherapy with and without lazertinib in EGFR-mutant advanced NSCLC after disease progression on osimertinib: primary results from the phase III MARIPOSA-2 study. Ann Oncol. (2024) 35:77–90. doi: 10.1016/j.annonc.2023.10.117, PMID: 37879444

[25] Zheng H Qin X Zheng Y Yang X Tan J Cai W . Addition of bevacizumab to EGFR tyrosine kinase inhibitors in advanced NSCLC: an updated systematic review and meta-analysis. Front Pharmacol. (2023) 14:1238579. doi: 10.3389/fphar.2023.1238579, PMID: 38269283 PMC10807044

[26] Zhao Y He Y Wang W Cai Q Ge F Chen Z . Efficacy and safety of immune checkpoint inhibitors for individuals with advanced EGFR-mutated non-small-cell lung cancer who progressed on EGFR tyrosine-kinase inhibitors: a systematic review, meta-analysis, and network meta-analysis. Lancet Oncol. (2024) 25:1347–56. doi: 10.1016/S1470-2045(24)00379-6, PMID: 39159630

[27] Qian X Guo X Li T Hu W Zhang L Wu C . Efficacy of immune checkpoint inhibitors in EGFR-Mutant NSCLC patients with EGFR-TKI resistance: A systematic review and meta-analysis. Front Pharmacol. (2022) 13:926890. doi: 10.3389/fphar.2022.926890, PMID: 36071838 PMC9442341

[28] Liu N Zhou Y Lee JJ . IPDfromKM: reconstruct individual patient data from published Kaplan-Meier survival curves. BMC Med Res Methodol. (2021) 21:111. doi: 10.1186/s12874-021-01308-8, PMID: 34074267 PMC8168323

[29] Baik C Lee S Cook K Wallace S Wood R Santana-Davila R . A Phase II study of nab-Paclitaxel (nab-P) in patients with advanced non-small cell lung cancer with EGFR mutations after frontline tyrosine kinase inhibitor therapy. Cancer Treat Res Commun. (2021) 28:100416. doi: 10.1016/j.ctarc.2021.100416, PMID: 34118789

[30] Jiang T Wang P Zhang J Zhao Y Zhou J Fan Y . Toripalimab plus chemotherapy as second-line treatment in previously EGFR-TKI treated patients with EGFR-mutant-advanced NSCLC: a multicenter phase-II trial. Signal Transduct Target Ther. (2021) 6:355. doi: 10.1038/s41392-021-00751-9, PMID: 34650034 PMC8517012

[31] Lam TC Tsang KC Choi HC Lee VH Lam KO Chiang CL . Combination atezolizumab, bevacizumab, pemetrexed and carboplatin for metastatic EGFR mutated NSCLC after TKI failure. Lung Cancer. (2021) 159:18–26. doi: 10.1016/j.lungcan.2021.07.004, PMID: 34303276

[32] Watanabe S Furuya N Nakamura A Shiihara J Nakachi I Tanaka H . A phase II study of atezolizumab with bevacizumab, carboplatin, and paclitaxel for patients with EGFR-mutated NSCLC after TKI treatment failure (NEJ043 study). Eur J Cancer. (2024) 197:113469. doi: 10.1016/j.ejca.2023.113469, PMID: 38061214

[33] Park S Kim TM Han JY Lee GW Shim BY Lee YG . Randomized study of atezolizumab plus bevacizumab and chemotherapy in patients with EGFR- or ALK-mutated non-small-cell lung cancer (ATTLAS, KCSG-LU19-04). J Clin Oncol. (2024) 42:1241–51. doi: 10.1200/JCO.23.01891, PMID: 37861993 PMC11095857

[34] Reck M Mok TSK Nishio M Jotte RM Cappuzzo F Orlandi F . Atezolizumab plus bevacizumab and chemotherapy in non-small-cell lung cancer (IMpower150): key subgroup analyses of patients with EGFR mutations or baseline liver metastases in a randomised, open-label phase 3 trial. Lancet Respir Med. (2019) 7:387–401. doi: 10.1016/S2213-2600(19)30084-0, PMID: 30922878

[35] Qin BD Jiao XD Yuan LY Wu Y Ling Y Zang YS . Immunotherapy-based regimens for patients with EGFR-mutated non-small cell lung cancer who progressed on EGFR-TKI therapy. J Immunother Cancer. (2024) 12. doi: 10.1136/jitc-2024-008818, PMID: 38631713 PMC11029279

[36] Riley RD Lambert PC Abo-Zaid G . Meta-analysis of individual participant data: rationale, conduct, and reporting. Bmj. (2010) 340:c221. doi: 10.1136/bmj.c221, PMID: 20139215

[37] Stewart LA Tierney JF . To IPD or not to IPD? Advantages and disadvantages of systematic reviews using individual patient data. Eval Health Prof. (2002) 25:76–97. doi: 10.1177/0163278702025001006, PMID: 11868447

[38] Yu HA Goto Y Hayashi H Felip E Chih-Hsin Yang J Reck M . HERTHENA-lung01, a phase II trial of patritumab deruxtecan (HER3-DXd) in epidermal growth factor receptor-mutated non-small-cell lung cancer after epidermal growth factor receptor tyrosine kinase inhibitor therapy and platinum-based chemotherapy. J Clin Oncol. (2023) 41:5363–75. doi: 10.1200/JCO.23.01476, PMID: 37689979 PMC10713116

[39] Yu HA Baik C Kim DW Johnson ML Hayashi H Nishio M . Translational insights and overall survival in the U31402-A-U102 study of patritumab deruxtecan (HER3-DXd) in EGFR-mutated NSCLC. Ann Oncol. (2024) 35:437–47. doi: 10.1016/j.annonc.2024.02.003, PMID: 38369013

[40] Paz-Ares L Ahn MJ Lisberg AE Kitazono S Cho BC Blumenschein G . 1314MO TROPION-Lung05: Datopotamab deruxtecan (Dato-DXd) in previously treated non-small cell lung cancer (NSCLC) with actionable genomic alterations (AGAs). Ann Oncol. (2023) 34:S755–S6. doi: 10.1016/j.annonc.2023.09.2348

