# Supplementary material for: Efficacy and safety analysis of treatment in patients with EGFR-mutated advanced NSCLC who progressed on TKIs: a systematic review and meta-analysis
Source: Front Immunol. 2025 Oct 28;16:1673115. doi: 10.3389/fimmu.2025.1673115 (PMC12602472; doi:10.3389/fimmu.2025.1673115)
Supplement: Supplementary file 1 [file DataSheet1.docx]

**Efficacy and Safety Analysis of Treatment in Patients with** **EGFR-mutated Advanced NSCLC Who Progressed on TKI: A Systematic Review and Meta-Analysis**

Supplementary Materials

| **Table of Contents** | | |
| --- | --- | --- |
| Title | Content | Page |
| Table S1 | Characteristics of Included Studies | 2-3 |
| Table S2 | Search Strategy and Data Extraction | 4-5 |
| Table S3 | Risk of bias assessment according to MINORS criteria | 6 |
| Table S4 | Checklist of the PRISMA extension for network meta-analysis | 7-13 |
| Table S5 | Comparison of the fit goodness between consistency and inconsistency models based on DIC values in network meta-analysis. | 14 |
| Table S6 | Sensitivity analysis of IPD meta-analysis: inclusion and exclusion of single-arm trials | 15 |
| Figure S1 | Heterogeneity based on PFS | 16 |
| Figure S2 | Heterogeneity based on OS | 17 |
| Figure S3 | Heterogeneity based on ORR | 18 |
| Figure S4 | Heterogeneity based on AE | 19 |
| Figure S5 | Risk of Bias Summary | 20 |
| Figure S6 | Risk of Bias Graph | 21 |
| Figure S7 | Subgroup analysis of treatment effects by prior TKI generation (1st/2nd vs. 3rd generation) in network meta-analysis: ICI-chemo vs chemo. | 22 |
| Figure S8 | Subgroup analysis of treatment effects by EGFR mutation subtype (L858R vs. 19DEL) in network meta-analysis: a. ICI-chemo vs chemo b. ICI-chemo-antiangio vs chemo. | 23 |
| Figure S9 | Subgroup analysis of treatment effects by EGFR mutation subtype (T790M+ vs. T790M-) in network meta-analysis: a. ICI-chemo vs chemo b. ICI-chemo-antiangio vs chemo. | 24 |
| Figure S10 | Subgroup analysis of treatment effects by brain metastasis status (With vs. Without) in network meta-analysis: ICI-chemo vs chemo. | 25 |
| Figure S11 | Trace and posterior density plots for treatment effect parameters in the Bayesian network meta-analysis. | 26 |
| Figure S12 | Gelman-Rubin diagnostic plots for convergence assessment in the Bayesian network meta-analysis. | 27 |

Table S1. Characteristics of Included Studies

|  | Study | | Single arm/ random | Phase  2/3 | Treatment | group | No of patients | PFS，  m | OS,  m | ORR |
| --- | --- | --- | --- | --- | --- | --- | --- | --- | --- | --- |
| 1 | | NEJ043 study | Single arm | 2 | atezolizumab,bevacizumab, carboplatin, paclitaxel | ICI-chemo-antiangio | 60 | 7.4 | 23.1 | 55.9% |
| 2 | | NCT03647956 | Single arm | 2 | atezolizumab, bevacizumab, pemetrexed and carboplatin | ICI-chemo-antiangio | 40 | 9.4 | NR | 62.5%% |
| 3 | | NCT01620190 | Single arm | 2 | nab-Paclitaxel | chemo | 26 | 4.0 | 9.0 | 35% |
| 4 | | ILLUMINATE | Single arm | 2 | durvalumab +tremelimumab+carboplatin‐pemetrexed | ICI-chemo | 100 |  |  | 26% |
| 5 | | NCT03924050 | Single arm | 2 | Toripalimab plus carboplatin and pemetrexed | ICI-chemo | 40 | 7.0 | 23.5 | 50% |
| 6 | | APPLE | random | 3 | atezolizumab+carboplatin+pemetrexed | ICI-chemo | 52 | 5.7 | 20.5 |  |
|  | |  |  |  | atezolizumab+carboplatin+pemetrexed+bevacizumab | ICI-chemo-antiangio | 52 | 9.6 | 31.4 |  |
| 7 | | ATTLAS | random | 3 | pemetrexed plus carboplatin or cisplatin | chemo | 68 | 5.62 | 20.27 |  |
|  | |  |  |  | atezolizumab + bevacizumab+paclitaxel+ carboplatin | ICI-chemo-antiangio | 147 | 8.71 | 20.6 |  |
| 8 | | CheckMate 722 | random | 3 | platinum-doublet chemotherapy | chemo | 150 | 5.4 | 15.9 | 26.7% |
|  | |  |  |  | nivolumab+ platinum-doublet chemotherapy | ICI-chemo | 144 | 5.6 | 19.4 | 31.3% |
| 9 | | MARIPOSA-2 | random | 3 | amivantamab-lazertinib-chemotherapy | amiva-lazer-chemo | 263 | 8.3 |  | 63% |
|  | |  |  |  | amivantamab+chemotherapy | amiva-chemo | 131 | 6.3 |  | 64% |
|  | |  |  |  | chemotherapy alone | chemo | 263 | 4.2 |  | 36% |
| 10 | | IMpower150 | random | 3 | Atezolizumab+Bevacizumab+Chemotherapy | ICI-chemo-antiangio | 34 | 10.2 | 26.1 | 70.6% |
|  | |  |  |  | Atezolizumab +carboplatin+ paclitaxel | ICI-chemo | 45 | 6.9 | 21.4 | 35.6% |
|  | |  |  |  | bevacizumab +carboplatin and paclitaxel | chemo-antiangio | 44 | 6.9 | 20.3 | 41.9% |
| 11 | | KEYNOTE789 | random | 3 | pembrolizumab+ chemotherapy | ICI-chemo | 245 | 5.6 | 15.9 | 29% |
|  | |  |  |  | placebo+chemotherapy | chemo | 247 | 5.5 | 14.7 | 27.1% |
| 12 | | HARMONi-A | random | 3 | AK112+chemotherapy | AK112+chemo | 161 | 7.1 | NR | 50.6% |
|  | |  |  |  | Placebo+chemotherapy | chemo | 161 | 4.8 | 14.3 | 35.4% |
| 13 | | ORINTAL-31 | random | 3 | Sintilimab +IBI305 +chemotherapy | ICI-chemo-antiangio | 158 | 7.2 | 21.1 | 48.1% |
|  | |  |  |  | Sintilimab +chemotherapy | ICI-chemo | 158 | 5.5 | 20.5 | 35% |
|  | |  |  |  | chemotherapy | chemo | 160 | 4.3 | 19.2 | 29% |
| 14 | | IMpower151 | random | 3 | atezolizumab + bevacizumab + carboplatin + pemetrexed or Pac | ICI-chemo-antiangio | 81 | 8.5 |  |  |
|  | |  |  |  | placebo + bevacizumab + carboplatin + pemetrexed or Pac | chemo-antiangio | 82 | 8.3 |  |  |

Table S2. Search Strategy and Data Extraction

| **Search Strategy** |
| --- |
| ((((((((((((((non-small-cell lung cancer[Title]) OR (non-small cell lung cancer[Title])) OR (non small-cell lung cancer[Title])) OR (non small cell lung cancer[Title])) OR (non-small-cell lung carcinoma[Title])) OR (non-small cell lung carcinoma[Title])) OR (non small-cell lung carcinoma[Title])) OR (non small cell lung carcinoma[Title])) OR (nsclc[Title])) AND (((((((((((((((((((EGFR-mutant[Title/Abstract]) OR (EGFR-mutated[Title/Abstract])) OR (EGFR-activating mutations[Title/Abstract])) OR (EGFR mutant[Title/Abstract])) OR (Epidermal Growth Factor Receptor Mutant[Title/Abstract])) ) OR (EGFR activating mutations[Title/Abstract])) OR (EGFR-Sensitive Mutations[Title/Abstract])) OR (Exon19-del[Title/Abstract])) OR (EGFR mutations [Title/Abstract])) OR (exon 19 deletions[Title/Abstract])) OR (exon 21[Title/Abstract])) OR (Leu858Arg[Title/Abstract])) OR (L858R[Title/Abstract])) OR (EGFR common mutations[Title/Abstract])) OR (common EGFR mutations[Title/Abstract]) ) OR (EGFR Variant[Title/Abstract])) OR (genetic driver alterations[Title/Abstract])) OR (EGFR mutation[Title/Abstract]))) AND (((((((((Progression[Title/Abstract]) OR (resistance[Title/Abstract])) OR (Previously Treated[Title/Abstract])) OR (TKI failure[Title/Abstract])) OR (post-TKI[Title/Abstract]) ) OR (disease progressed[Title/Abstract])) OR (pretreatment[Title/Abstract])) OR (pretreated[Title/Abstract])) OR (received treatment[Title/Abstract]))) AND (((((((Randomized Controlled Trial[Publication Type]) OR (controlled clinical trial[Publication Type])) OR (randomized[Title/Abstract])) OR (randomised[Title/Abstract])) OR (randomly[Title/Abstract])) OR (trial[Title/Abstract])) OR (phase[Title/Abstract]))) AND ((((((((((((((((((((((((((((((((((((((((((((((((immunotherapy[Title/Abstract]) OR (immune checkpoint inhibitor[Title/Abstract])) OR (programmed cell death protein 1[Title/Abstract])) OR (Programmed death ligand-1[Title/Abstract])) OR (PD-1[Title/Abstract])) OR (PD-L1[Title/Abstract])) OR (cytotoxic T-lymphocyte-associated protein 4[Title/Abstract])) OR (CTLA-4[Title/Abstract])) OR (Nivolumab[Title/Abstract])) OR (Opdivo[Title/Abstract])) OR (Pembrolizumab[Title/Abstract])) OR (Keytruda[Title/Abstract])) OR (MK-3475[Title/Abstract])) OR (Atezolizumab[Title/Abstract])) OR (Tecentrip[Title/Abstract])) OR (MPDL3280A[Title/Abstract])) OR (Durvalumab[Title/Abstract])) OR (Imfinzi[Title/Abstract])) OR (MEDI 4736[Title/Abstract])) OR (ipilimumab[Title/Abstract])) OR (tremelimumab[Title/Abstract])) OR (camrelizumab[Title/Abstract])) OR (sintilimab[Title/Abstract])) OR (tislelizumab[Title/Abstract])) OR (toripalimab[Title/Abstract])) OR (cemiplimab[Title/Abstract])) OR (sugemalimab[Title/Abstract])) OR (avelumab[Title/Abstract])) OR (serplulimab[Title/Abstract])) OR (TQB2450[Title/Abstract])) OR (envafolimab[Title/Abstract])) OR (zimberelimab[Title/Abstract])) OR (pucotenlimab[Title/Abstract])) OR (penpulimab[Title/Abstract])) OR (cadonilimab[Title/Abstract])) OR (AK104[Title/Abstract])) OR (relatlimab[Title/Abstract])) OR (dostarlimab[Title/Abstract])) OR (AK112[Title/Abstract])) OR (Ivonescimab[Title/Abstract])) OR (PM8002[Title/Abstract])) OR ((((((((((((((((antibody drug conjugate[Title/Abstract]) OR (ADC[Title/Abstract])) OR (TROP2 ADC[Title/Abstract])) OR (Dato-DXd[Title/Abstract])) OR (Datopotamab deruxtecan[Title/Abstract])) OR (sacituzumab govitecan[Title/Abstract])) OR (SKB264[Title/Abstract])) OR (Sacituzumab tirumotecan[Title/Abstract])) OR (HER3 ADC[Title/Abstract])) OR (HER3-DXd[Title/Abstract])) OR (patritumab deruxtecan[Title/Abstract])) OR (BL-B01D1[Title/Abstract])) OR (YL202[Title/Abstract])) OR (SHR-A2009[Title/Abstract])) OR (DS-1062a[Title/Abstract])) OR (IMMU-132[Title/Abstract]))) OR ((((((((((((((((Angiogenesis[Title/Abstract]) OR (anti-VEGF therapy[Title/Abstract])) OR (bevacizumab[Title/Abstract])) OR (ramucirumab[Title/Abstract])) OR (anti-angiogenic agents[Title/Abstract])) OR (anlotinib[Title/Abstract])) OR (apatinib[Title/Abstract])) OR (lenvatinib[Title/Abstract])) OR (small molecule VEGF signaling pathway inhibitors[Title/Abstract])) OR (Nintedanib[Title/Abstract])) OR (Sunitinib[Title/Abstract])) OR (Pazopanib[Title/Abstract])) OR (Axitinib[Title/Abstract])) OR (Cediranib[Title/Abstract])) OR (Motesanib[Title/Abstract])) OR (BAT1706[Title/Abstract]))) OR ((chemotherapy[Title/Abstract]) OR (docetaxel[Title/Abstract]))))) OR (Pemetrexed[Title/Abstract])) OR (paclitaxel[Title/Abstract]))) AND (English[Language])) AND (("2018/04/19"[Date - Publication] : "2024/06/30"[Date - Publication])) |

Table S3. Risk of bias assessment according to MINORS criteria.

| Study | 1. A clearly stated aim | 2. Inclusion of consecutive patients | 3. Prospective collection of data | 4. Endpoints appropriate to the aim | 5. Unbiased assessment of endpoint | 6. Follow-up period appropriate | 7. Loss to follow up less than 5% | 8. Prospective calculation of size | Quality score (max 16) |
| --- | --- | --- | --- | --- | --- | --- | --- | --- | --- |
| NEJ043 study | 2 | 2 | 2 | 2 | 1 | 2 | 2 | 2 | 15 |
| NCT03647956 | 2 | 2 | 2 | 2 | 1 | 2 | 2 | 1 | 14 |
| NCT01620190 | 2 | 2 | 2 | 2 | 1 | 2 | 2 | 1 | 14 |
| ILLUMINATE | 2 | 1 | 2 | 2 | 1 | 1 | 1 | 2 | 12 |
| NCT03924050 | 2 | 1 | 2 | 2 | 1 | 1 | 1 | 2 | 12 |

Table S4. Checklist of the PRISMA extension for network meta-analysis

| **Section and Topic** | **Item #** | **Checklist item** | **Location where item is reported** |
| --- | --- | --- | --- |
| **TITLE** | | |  |
| Title | 1 | Identify the report as a systematic review. | Page 1, line 1 |
| **ABSTRACT** | | |  |
| Abstract | 2 | See the PRISMA 2020 for Abstracts checklist. | Page 2, line 17-50 |
| **INTRODUCTION** | | |  |
| Rationale | 3 | Describe the rationale for the review in the context of existing knowledge. | Page 4, line 54-115 |
| Objectives | 4 | Provide an explicit statement of the objective(s) or question(s) the review addresses. | Page 6, line 116-126 |
| **METHODS** | | |  |
| Eligibility criteria | 5 | Specify the inclusion and exclusion criteria for the review and how studies were grouped for the syntheses. | Page 8, line 142-148 |
| Information sources | 6 | Specify all databases, registers, websites, organisations, reference lists and other sources searched or consulted to identify studies. Specify the date when each source was last searched or consulted. | Page 7, line 134-140 |
| Search strategy | 7 | Present the full search strategies for all databases, registers and websites, including any filters and limits used. | Page 7, line 134-140 |
| Selection process | 8 | Specify the methods used to decide whether a study met the inclusion criteria of the review, including how many reviewers screened each record and each report retrieved, whether they worked independently, and if applicable, details of automation tools used in the process. | Page 8, line 150-161 |
| Data collection process | 9 | Specify the methods used to collect data from reports, including how many reviewers collected data from each report, whether they worked independently, any processes for obtaining or confirming data from study investigators, and if applicable, details of automation tools used in the process. | Page 8, line 150-161 |
| Data items | 10a | List and define all outcomes for which data were sought. Specify whether all results that were compatible with each outcome domain in each study were sought (e.g. for all measures, time points, analyses), and if not, the methods used to decide which results to collect. | Page 8, line 150-161 |
|  | 10b | List and define all other variables for which data were sought (e.g. participant and intervention characteristics, funding sources). Describe any assumptions made about any missing or unclear information. | Page 8, line 150-161 |
| Study risk of bias assessment | 11 | Specify the methods used to assess risk of bias in the included studies, including details of the tool(s) used, how many reviewers assessed each study and whether they worked independently, and if applicable, details of automation tools used in the process. | Page 8, line 158-161 |
| Effect measures | 12 | Specify for each outcome the effect measure(s) (e.g. risk ratio, mean difference) used in the synthesis or presentation of results. | Page 9, line 163-173 |
| Synthesis methods | 13a | Describe the processes used to decide which studies were eligible for each synthesis (e.g. tabulating the study intervention characteristics and comparing against the planned groups for each synthesis (item #5)). | Page 8, line 142-148 |
|  | 13b | Describe any methods required to prepare the data for presentation or synthesis, such as handling of missing summary statistics, or data conversions. | Page 9, line 163-184 |
|  | 13c | Describe any methods used to tabulate or visually display results of individual studies and syntheses. | Page 9, line 163-184 |
|  | 13d | Describe any methods used to synthesize results and provide a rationale for the choice(s). If meta-analysis was performed, describe the model(s), method(s) to identify the presence and extent of statistical heterogeneity, and software package(s) used. | Page 9, line 163-184 |
|  | 13e | Describe any methods used to explore possible causes of heterogeneity among study results (e.g. subgroup analysis, meta-regression). | Page 9, line 181-184 |
|  | 13f | Describe any sensitivity analyses conducted to assess robustness of the synthesized results. | Page 9, line 163-184 |
| Reporting bias assessment | 14 | Describe any methods used to assess risk of bias due to missing results in a synthesis (arising from reporting biases). | Page 9, line 163-184 |
| Certainty assessment | 15 | Describe any methods used to assess certainty (or confidence) in the body of evidence for an outcome. | Page 9, line 163-184 |
| **RESULTS** | | |  |
| Study selection | 16a | Describe the results of the search and selection process, from the number of records identified in the search to the number of studies included in the review, ideally using a flow diagram. | Page 10, line 188-193 |
|  | 16b | Cite studies that might appear to meet the inclusion criteria, but which were excluded, and explain why they were excluded. | Page 10, line 188-193 |
| Study characteristics | 17 | Cite each included study and present its characteristics. | Page 10, line 188-193 |
| Risk of bias in studies | 18 | Present assessments of risk of bias for each included study. | Page 11, line 239-246 |
| Results of individual studies | 19 | For all outcomes, present, for each study: (a) summary statistics for each group (where appropriate) and (b) an effect estimate and its precision (e.g. confidence/credible interval), ideally using structured tables or plots. | Page 12, line 238-245 |
| Results of syntheses | 20a | For each synthesis, briefly summarise the characteristics and risk of bias among contributing studies. | Page 12, line 238-245 |
|  | 20b | Present results of all statistical syntheses conducted. If meta-analysis was done, present for each the summary estimate and its precision (e.g. confidence/credible interval) and measures of statistical heterogeneity. If comparing groups, describe the direction of the effect. | Page 10, line 194-210 |
|  | 20c | Present results of all investigations of possible causes of heterogeneity among study results. | Page 11, line 211-215 |
|  | 20d | Present results of all sensitivity analyses conducted to assess the robustness of the synthesized results. | Page 11, line 211-215 |
| Reporting biases | 21 | Present assessments of risk of bias due to missing results (arising from reporting biases) for each synthesis assessed. | Page 12, line 238-245 |
| Certainty of evidence | 22 | Present assessments of certainty (or confidence) in the body of evidence for each outcome assessed. | Page 11, line 216-237 |
| **DISCUSSION** | | |  |
| Discussion | 23a | Provide a general interpretation of the results in the context of other evidence. | Page 12, line 247-330 |
|  | 23b | Discuss any limitations of the evidence included in the review. | Page 17, line 331-345 |
|  | 23c | Discuss any limitations of the review processes used. | Page 17, line 331-345 |
|  | 23d | Discuss implications of the results for practice, policy, and future research. | Page 17, line 351-359 |
| **OTHER INFORMATION** | | |  |
| Registration and protocol | 24a | Provide registration information for the review, including register name and registration number, or state that the review was not registered. | Page 7, line 129-132 |
|  | 24b | Indicate where the review protocol can be accessed, or state that a protocol was not prepared. | Page 7, line 129-132 |
|  | 24c | Describe and explain any amendments to information provided at registration or in the protocol. | Page 7, line 129-132 |
| Support | 25 | Describe sources of financial or non-financial support for the review, and the role of the funders or sponsors in the review. | Page 18, line 368-370 |
| Competing interests | 26 | Declare any competing interests of review authors. | Page 18, line 372-374 |
| Availability of data, code and other materials | 27 | Report which of the following are publicly available and where they can be found: template data collection forms; data extracted from included studies; data used for all analyses; analytic code; any other materials used in the review. | Page 7, line 133-140 |

Table S5. Comparison of the fit goodness between consistency and inconsistency models based on DIC values in network meta-analysis.

| Outcome | Model | DIC |
| --- | --- | --- |
| Progression-free survival | Consistency | 16.34 |
|  | Inconsistency | 19.66 |
| Overall survival | Consistency | 17.25 |
|  | Inconsistency | 18.3 |
| Objective response rate | Consistency | 28.53 |
|  | Inconsistency | 28.33 |
| Grade 3 or higher adverse events | Consistency | 30.02 |
|  | Inconsistency | 30.56 |

Table S6. Sensitivity analysis of IPD meta-analysis: inclusion and exclusion of single-arm trials

| Comparison | Subgroup | HR (95%CI) | P | N |
| --- | --- | --- | --- | --- |
| AK112_chemo vs ICI_chemo_antiangio | \| Excluding single-arm \| \| --- \| | 1.14 (0.91, 1.42) | 0.25 | 627 |
|  | Single-arm + RCT | 0.95(0.78,1.17) | 0.64 | 726 |
| amiva_lazer_chemo vs ICI_chemo_antiangio | Excluding single-arm | 0.91 (0.76, 1.08) | 0.26 | 729 |
|  | Single-arm + RCT | 0.88(0.75,1.04) | 0.15 | 828 |
| amiva_chemo vs ICI_chemo_antiangio | Excluding single-arm | 1.42 (1.15, 1.76) | < 0.001 | 598 |
|  | Single-arm + RCT | 1.27(1.04,1.56) | 0.02 | 696 |
| chemo vs ICI_chemo_antiangio | Excluding single-arm | 1.94 (1.72, 2.18) | < 0.001 | 1514 |
|  | Single-arm + RCT | 1.89(1.69,2.1) | < 0.001 | 1639 |
| chemo_antiangio vs ICI_chemo_antiangio | Excluding single-arm | 1.27 (1.03, 1.55) | 0.02 | 594 |
|  | Single-arm + RCT | 1.21(1,1.48) | 0.06 | 692 |
| ICI_chemo vs ICI_chemo_antiangio | Excluding single-arm | 1.47 (1.3, 1.67) | < 0.001 | 1110 |
|  | Single-arm + RCT | 1.41(1.26,1.58) | < 0.001 | 1347 |
| AK112_chemo vs chemo | Excluding single-arm | 0.52 (0.43, 0.63) | < 0.001 | 1209 |
|  | Single-arm + RCT | 0.50(0.42,0.61) | < 0.001 | 1235 |
| amiva_lazer_chemo vs chemo | Excluding single-arm | 0.48 (0.41, 0.57) | < 0.001 | 1311 |
|  | Single-arm + RCT | 0.47(0.4,0.55) | < 0.001 | 1337 |
| amiva_chemo vs chemo | Excluding single-arm | 0.66 (0.55, 0.8) | < 0.001 | 1179 |
|  | Single-arm + RCT | 0.67(0.55,0.82) | < 0.001 | 1205 |
| chemo_antiangio vs chemo | Excluding single-arm | 0.65 (0.53, 0.78) | < 0.001 | 1175 |
|  | Single-arm + RCT | 0.64(0.53,0.78) | < 0.001 | 1201 |
| ICI_chemo vs chemo | Excluding single-arm | 0.76 (0.68, 0.84) | < 0.001 | 1692 |
|  | Single-arm + RCT | 0.75(0.68,0.82) | < 0.001 | 1856 |
| ICI_chemo_antiangio vs chemo | Excluding single-arm | 0.52 (0.46, 0.58) | < 0.001 | 1514 |
|  | Single-arm + RCT | 0.53(0.48,0.59) | < 0.001 | 1639 |
|  |  |  |  |  |
|  |  |  |  |  |

Figure S1. Heterogeneity based on PFS


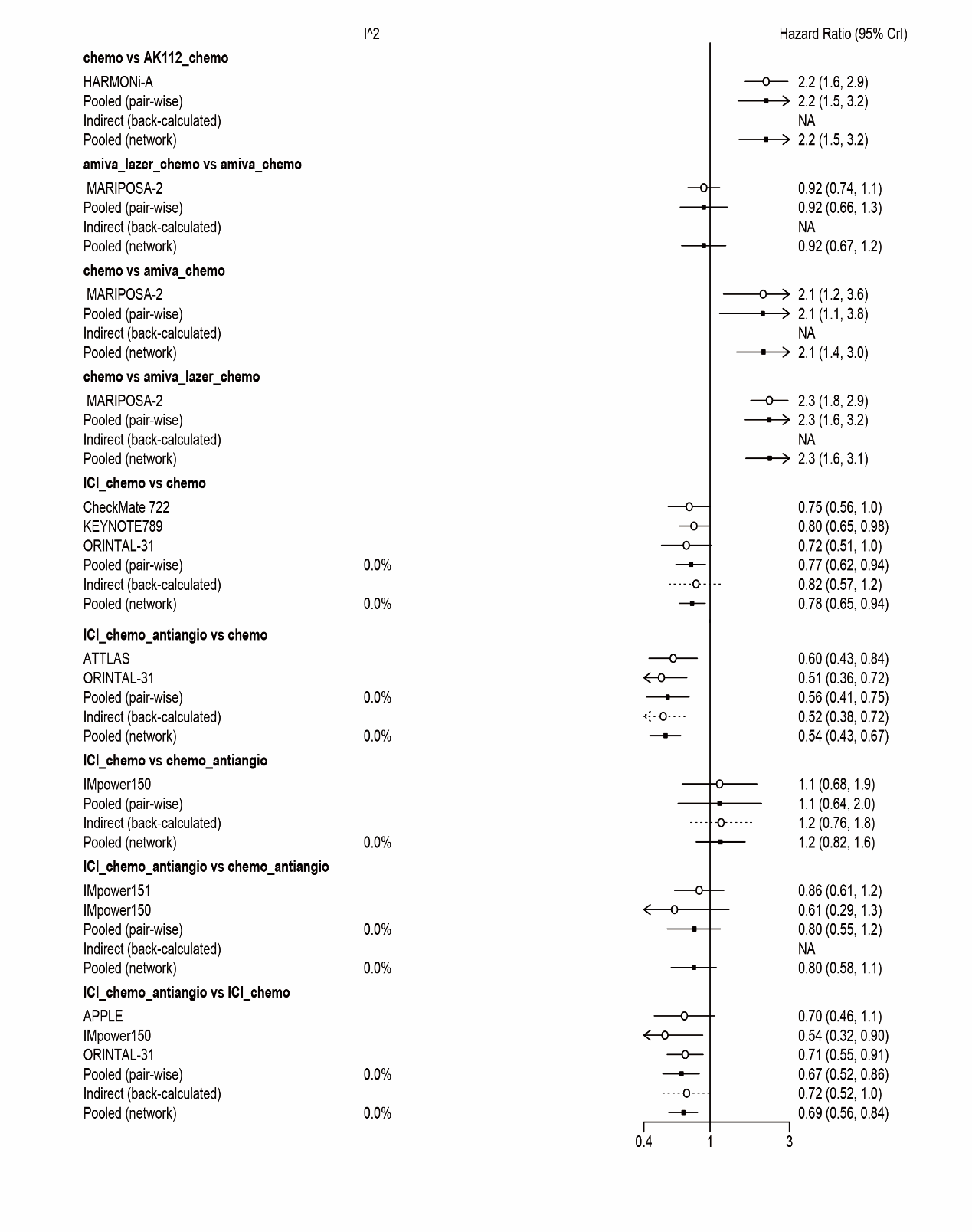


Figure S2. Heterogeneity based on OS


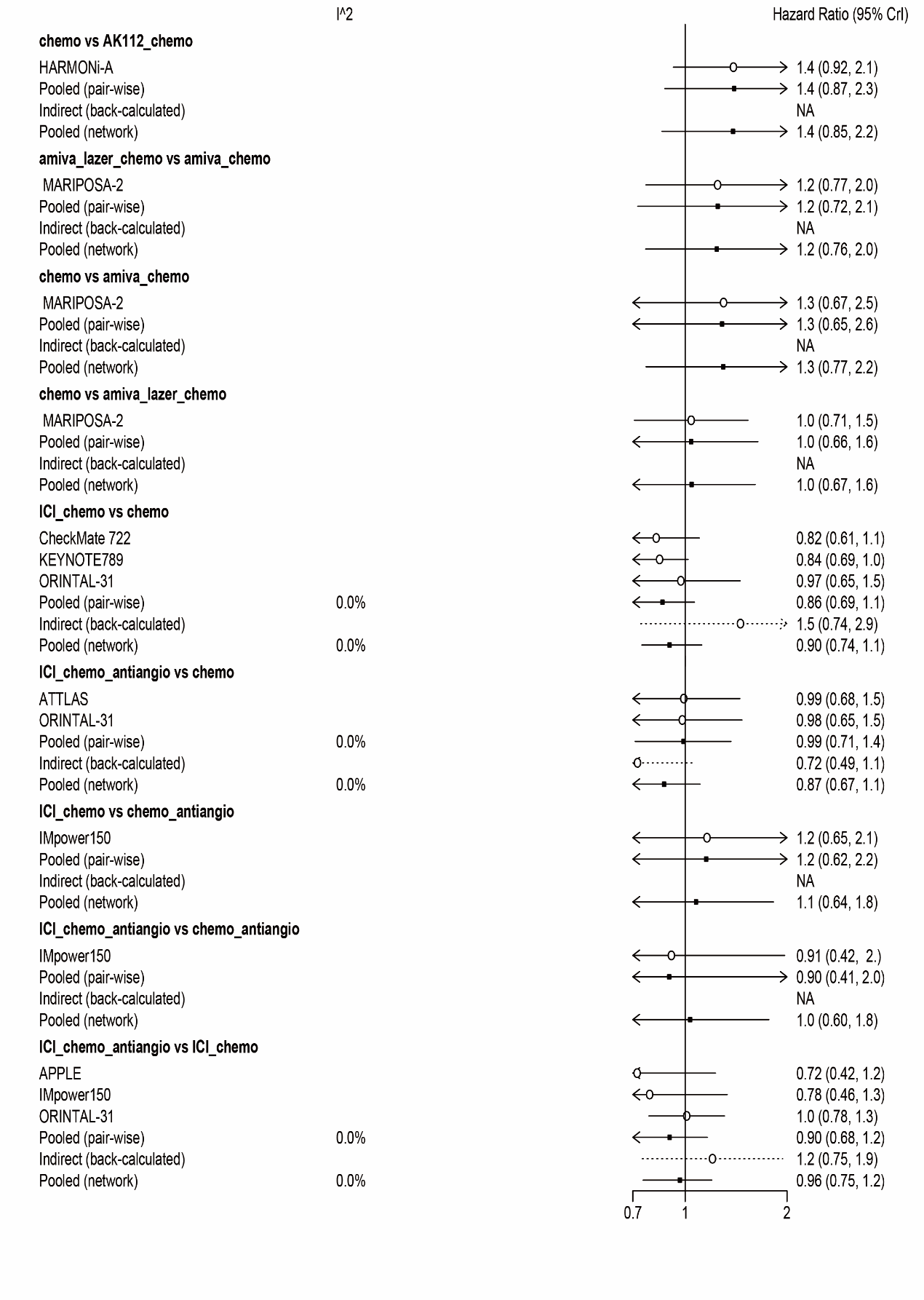


Figure S3. Heterogeneity based on ORR


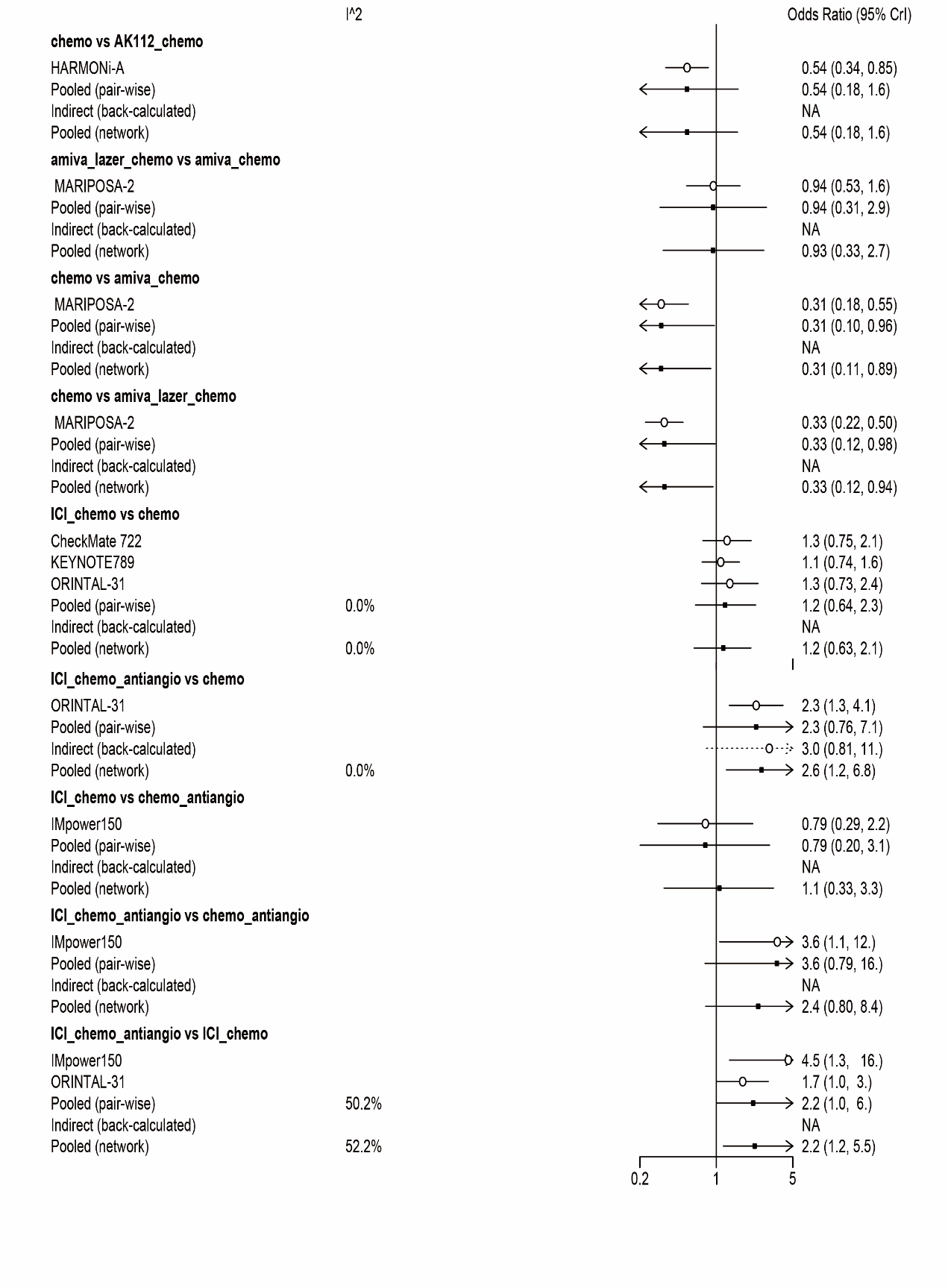


Figure S4. Heterogeneity based on AE


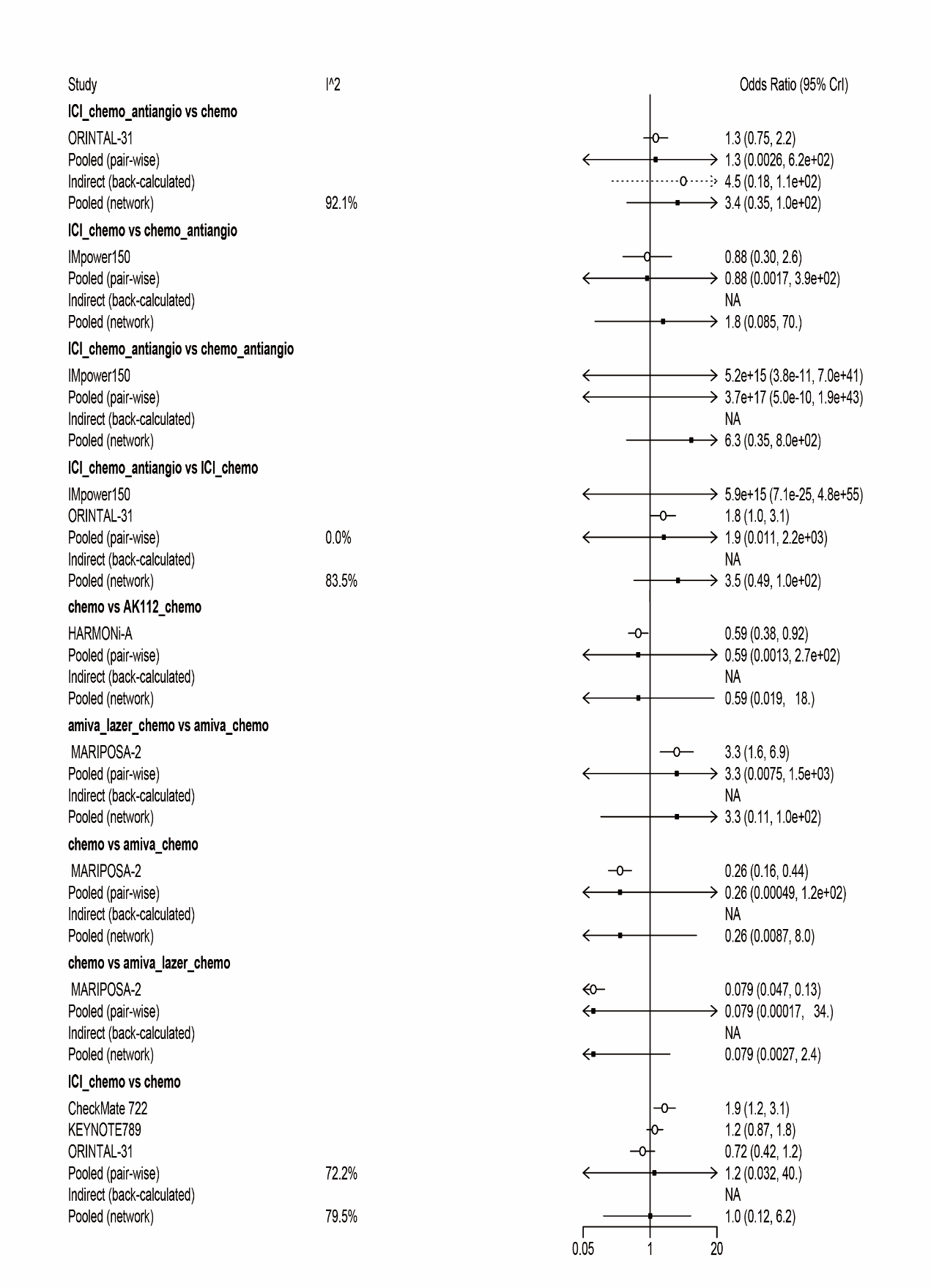


Figure S5. Risk of Bias Summary


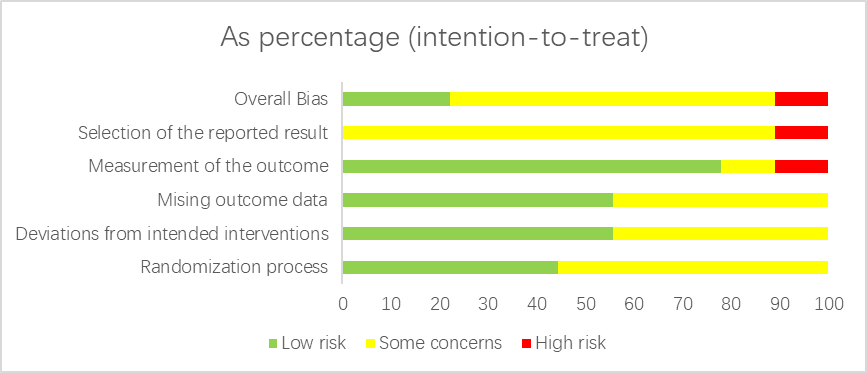

Figure S6. Risk of Bias Graph

Figure S7. Subgroup analysis of treatment effects by prior TKI generation (1st/2nd vs. 3rd generation) in network meta-analysis: ICI-chemo vs chemo.
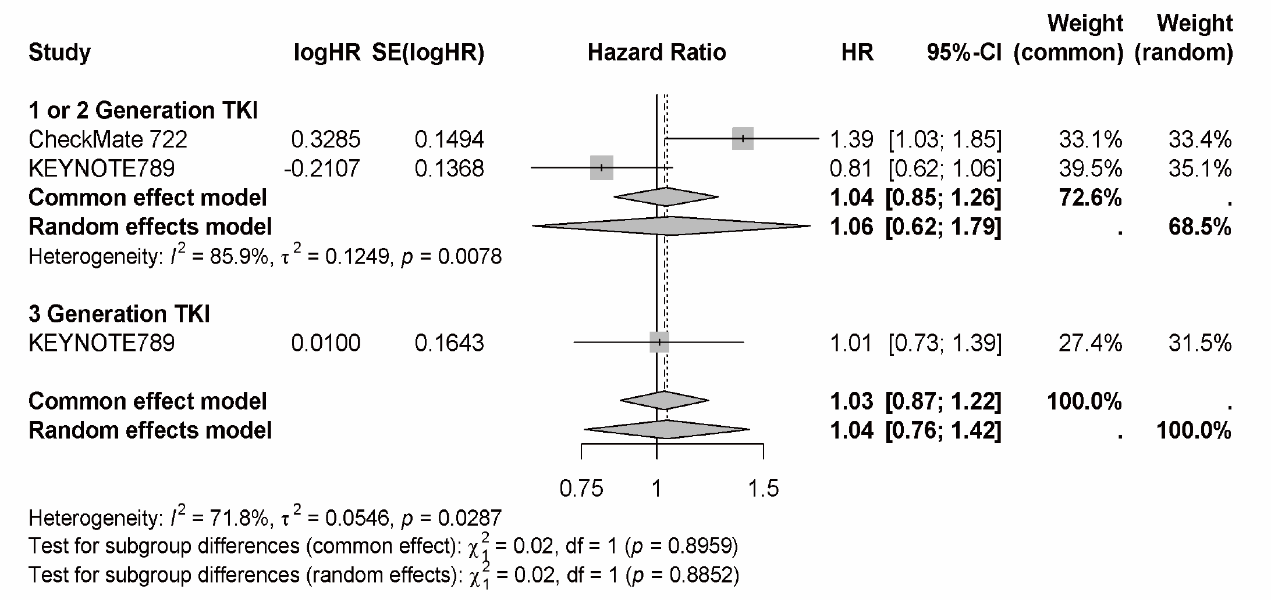


Figure S8. Subgroup analysis of treatment effects by EGFR mutation subtype (L858R vs. 19DEL) in network meta-analysis: a. ICI-chemo vs chemo b. ICI-chemo-antiangio vs chemo.
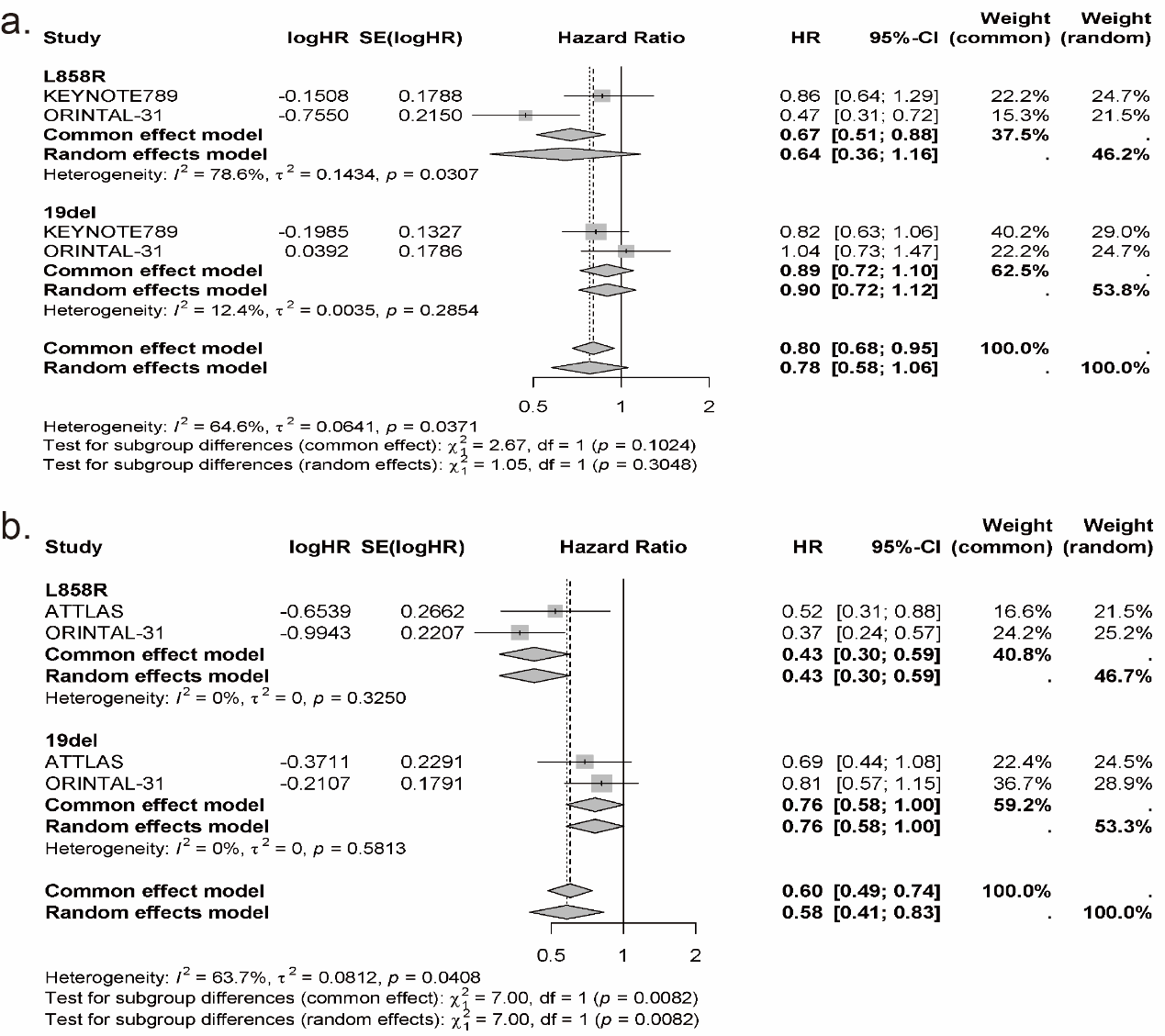


Figure S9. Subgroup analysis of treatment effects by EGFR mutation subtype (T790M+ vs. T790M-) in network meta-analysis: a. ICI-chemo vs chemo b. ICI-chemo-antiangio vs chemo.
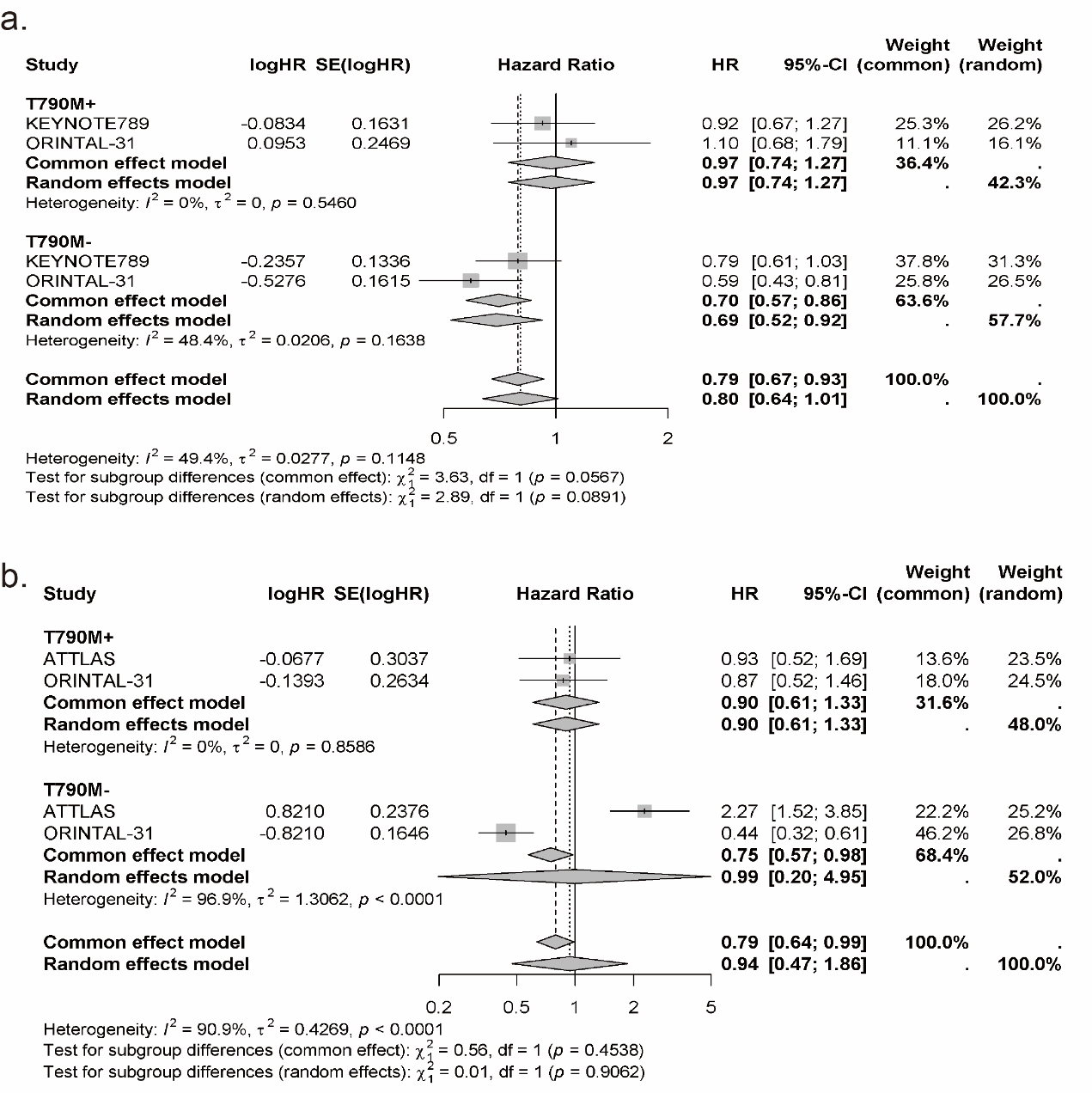


Figure S10. Subgroup analysis of treatment effects by brain metastasis status (With vs. Without) in network meta-analysis: ICI-chemo vs chemo.
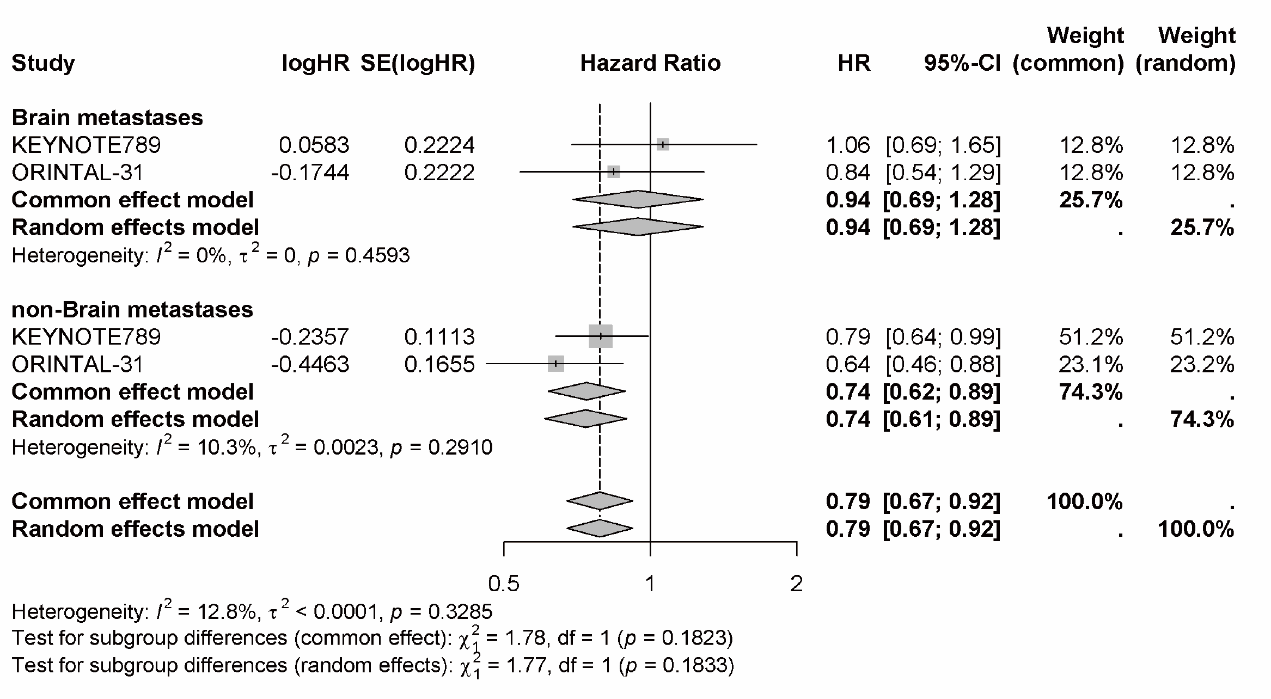


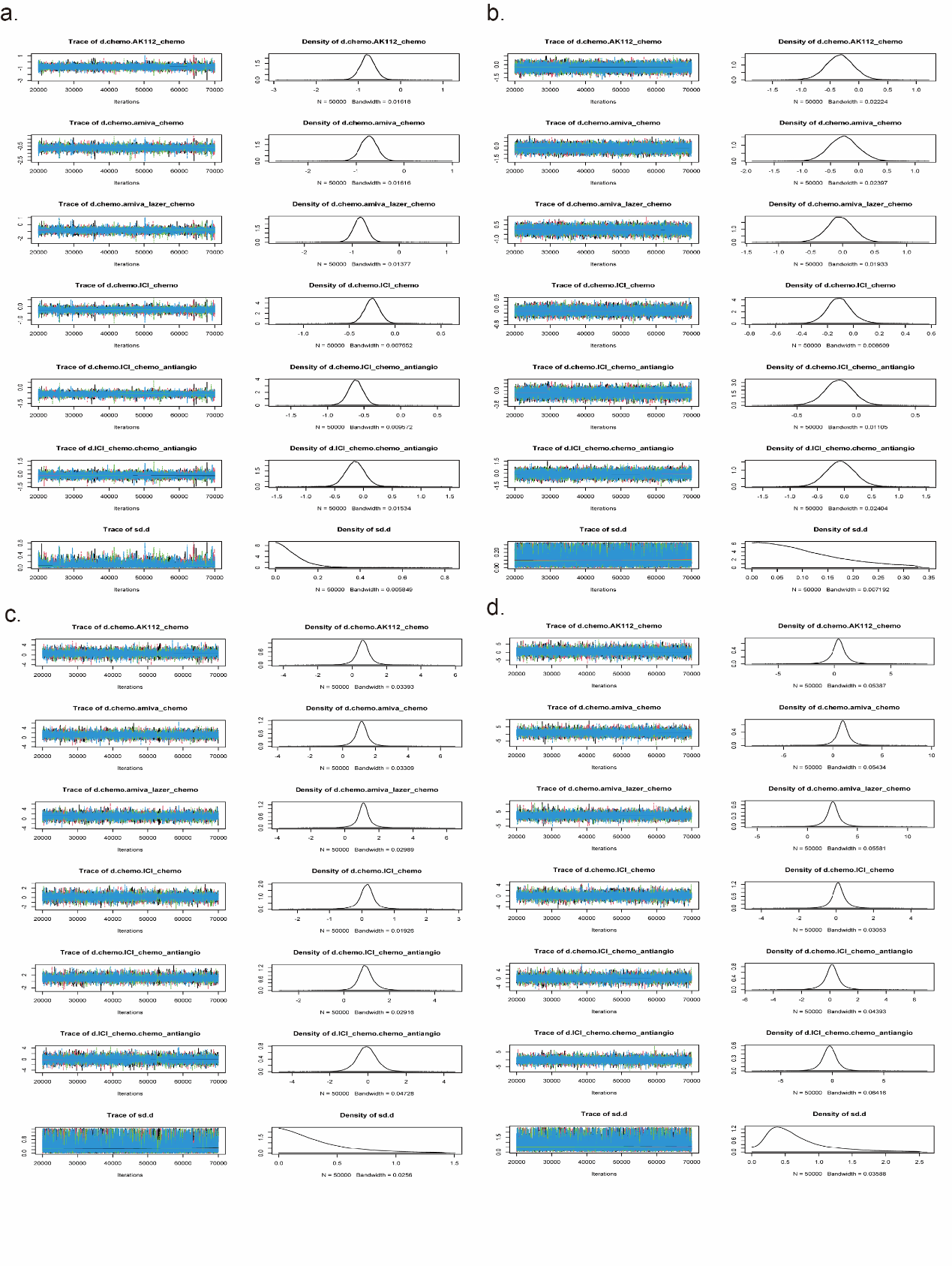
Figure S11. Trace and posterior density plots for treatment effect parameters in the Bayesian network meta-analysis.


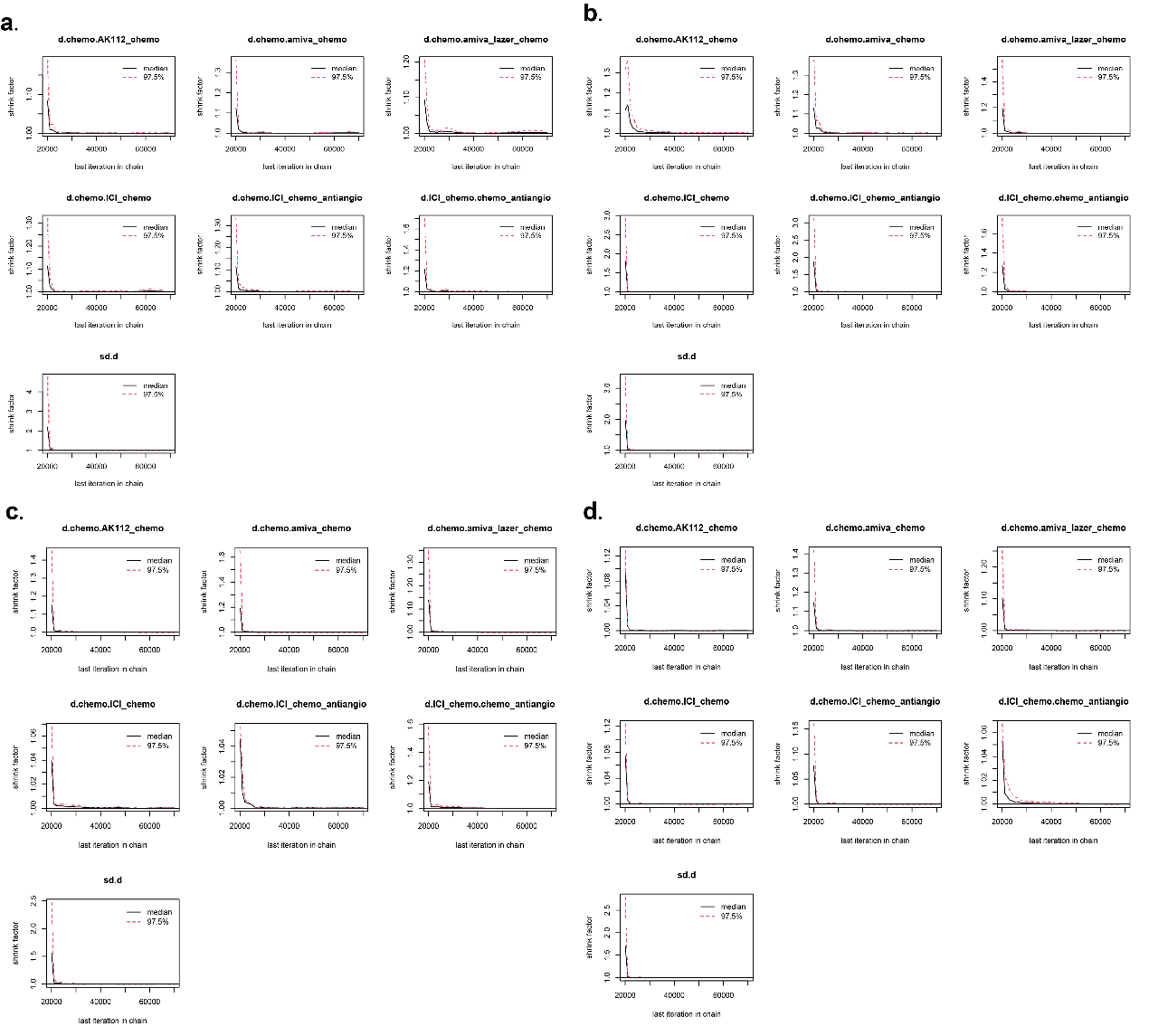
Figure S12. Gelman-Rubin diagnostic plots for convergence assessment in the Bayesian network meta-analysis.
